# FOXO regulates the expression of antimicrobial peptides and promotes phagocytosis of hemocytes in shrimp antibacterial immunity

**DOI:** 10.1371/journal.ppat.1009479

**Published:** 2021-04-02

**Authors:** Cang Li, Pan-Pan Hong, Ming-Chong Yang, Xiao-Fan Zhao, Jin-Xing Wang

**Affiliations:** 1 Shandong Provincial Key Laboratory of Animal Cells and Developmental Biology, School of Life Sciences, Shandong University, Qingdao, Shandong, China; 2 State Key Laboratory of Microbial Technology, Shandong University, Qingdao, Shandong, China; 3 Laboratory for Marine Biology and Biotechnology, Pilot National Laboratory for Marine Science and Technology (Qingdao), Qingdao, China; Duke University, UNITED STATES

## Abstract

Invertebrates rely on innate immunity, including humoral and cellular immunity, to resist pathogenic infection. Previous studies showed that forkhead box transcription factor O (FOXO) participates in mucosal immune responses of mammals and the gut humoral immune regulation of invertebrates. However, whether FOXO is involved in systemic and cellular immunity regulation in invertebrates remains unknown. In the present study, we identified a FOXO from shrimp (*Marsupenaeus japonicus)* and found that it was expressed at relatively basal levels in normal shrimp, but was upregulated significantly in shrimp challenged by *Vibrio anguillarum*. FOXO played a critical role in maintaining hemolymph and intestinal microbiota homeostasis by promoting the expression of *Relish*, the transcription factor of the immune deficiency (IMD) pathway for expression of antimicrobial peptides (AMPs) in shrimp. We also found that pathogen infection activated FOXO and induced its nuclear translocation by reducing serine/threonine kinase AKT activity. In the nucleus, activated FOXO directly regulated the expression of its target *Amp* and *Relish* genes against bacterial infection. Furthermore, FOXO was identified as being involved in cellular immunity by promoting the phagocytosis of hemocytes through upregulating the expression of the phagocytotic receptor scavenger receptor C (*Src*), and two small GTPases, *Rab5* and *Rab7*, which are related to phagosome trafficking to the lysosome in the cytoplasm. Taken together, our results indicated that FOXO exerts its effects on homeostasis of hemolymph and the enteric microbiota by activating the IMD pathway in normal shrimp, and directly or indirectly promoting AMP expression and enhancing phagocytosis of hemocytes against pathogens in bacteria-infected shrimp. This study revealed the different functions of FOXO in the mucosal (local) and systemic antibacterial immunity of invertebrates.

## Author summary

Shrimp aquaculture is one of the world’s fastest growing industries for producing animal proteins and has made a significant contribution to meeting the worldwide increased demand for animal proteins. However, disease outbreaks in aquaculture result in large economic losses to the industry. Studies of shrimp immune mechanisms could provide new strategies for disease prevention and control. The forkhead box transcription factor O family proteins (FOXOs) are involved in various critical biological process of organisms. However, whether FOXO is involved in systemic and cellular immunity regulation in invertebrates remains unknown. In the present study, we identified a FOXO from kuruma shrimp (*Marsupenaeus japonicus*) and found that it played a critical role in maintaining hemolymph and intestinal microbiota homeostasis by promoting the expression of *Relish*, the transcription factor of immune deficiency (IMD) pathway for expression of antimicrobial peptides (AMPs) in shrimp. We also found that pathogen infection activated FOXO and induced its nuclear translocation by reducing serine/threonine kinase AKT activity and directly regulated the expression of its target *Amp* and *Relish* genes against bacterial infection. Furthermore, FOXO was identified as being involved in cellular immunity by promoting the phagocytosis of hemocytes through upregulating the expression of the phagocytotic receptor scavenger receptor C (*Src*), and two small GTPases, *Rab5* and *Rab7*, which are related to phagosome trafficking to the lysosome in the cytoplasm. This study revealed the different functions of FOXO in the innate antibacterial immunity of invertebrates.

## Introduction

The forkhead box transcription factor O family proteins (FOXOs) are involved in various critical biological process of organisms, including cell cycle regulation, repair of damaged DNA, anti-cancer immunity, and life span regulation [1,2]. Mammals have four different FOXO proteins (FOXO1, FOXO3, FOXO4, and FOXO6); however, invertebrates have only one FOXO, which is also known as Daf-16 in *Caenorhabditis elegans* [3]. The transcriptional activity of FOXO is regulated by several kinds of post-translational modifications, such as phosphorylation, acetylation, methylation, and ubiquitination [4]. These modifications affect FOXO nuclear translocation or exit from the nucleus, and its interaction with co-repressors and co-activators, which can promote or decrease FOXO activity and mediate its different biological functions [5,6]. After activation, FOXO participates in many physiological functions by regulating the transcription of a variety of target genes [7].

FOXOs are activated by various extracellular stimuli, including growth factors, cytokines, and hormones [8]. Growth signals, such as insulin or insulin-like growth factor 1 (IGF-1), interact with receptors of the insulin pathway, and activate phosphatidylinositol 3-kinase (PI3K) signaling, leading to phosphorylation of FOXO by the serine/threonine kinase, protein kinase B (AKT) [9]. Phosphorylated FOXO translocates from the nucleus to the cytosol, or prevents its translocation from cytosol to the nucleus, where it becomes ubiquitinated, leading to its degradation by the proteasome [10]. By contrast, in the absence of external growth signals, the PI3K-AKT signaling pathway is inactive, and unphosphorylated FOXO translocates into the nucleus to promote target gene transcription [11]. FOXO also participates in the anti-bacterial and anti-virus innate immune responses of invertebrates [12,13]. Chronic activation of FOXO in the aging intestines of *Drosophila* represses the expression of peptidoglycan recognition protein SC2 (a negative regulator of the immune deficiency (IMD) pathway) and breaks intestinal immune homeostasis [14]. FOXO directly regulates antimicrobial peptide (AMP) genes in epithelial tissues of non-infected *Drosophila* in response to starvation stress, which is independent of pathogen-responsive innate immunity pathways [15]. However, pathogen infection can also induce enterocyte FOXO to enter the nucleus from cytoplasm directly in *Drosophila* intestines [12]. The FOXO-dependent regulation of AMPs also occurs in mammalian epithelial tissues, suggesting that this is evolutionarily conserved in animals [16].

Several studies showed that FOXOs are activated by pathogens or cytokine stimulation in mucosal immune responses [17]. Their translocation to the nucleus and binding to promoters of their target genes is stimulated by the mitogen-activated protein (MAP) kinase pathway and inhibited by the PI3K/AKT pathway. The target genes of FOXOs include pro-inflammatory signaling molecules, adhesion molecules, and chemokine receptors [17]. Whether FOXOs are involved in the systemic immune responses against pathogens remains unclear.

Promoting the expression of AMPs against pathogens is an important mechanism of humoral innate immunity in invertebrates [18]. In *Drosophila* innate immunity, the Toll and IMD pathways are involved in the regulation of AMP expression, and AMPs play important roles in the elimination of invading pathogens [19–21]. Similar to insect immunity, the Toll, IMD, and Janus kinase (JAK)/signal transducer and activator of transcription (STAT) pathways are involved in AMP expression regulation in crustaceans [22,23]. Whether FOXO can regulate the expression of AMPs independent of the Toll, IMD, and JAK/STAT pathways against pathogens infection in crustaceans requires clarification.

In addition to humoral immunity, invertebrates also rely on cellular immunity to perform innate immunity functions against pathogen infection. Phagocytosis by hemocytes is one of the important mechanism for cellular immunity against pathogen infection [24]. Previous studies have found that FOXO1-mediated autophagy is required for natural killer (NK) cell development [25]. Moreover, FOXO is involved in promoting bacterial phagocytosis by neutrophils [26]. Although there are no specialized immune cells in crustaceans, such as NK cell or neutrophils, shrimp hemocytes can exert systemic innate immunity against pathogenic bacteria infection via phagocytosis [27]. However, whether FOXO participates in the pathogen phagocytosis of hemocytes in shrimp is unclear.

Shrimp aquaculture is one of the world’s fastest growing industries for producing animal proteins and has made a significant contribution to meeting the worldwide increased demand for animal proteins. Currently, the global production of shrimp is approximately 4.88 million tons with a value of around 39 billion USD [28]. However, rearing large numbers of animals together results in substantial animal stress, which facilitates pathogen, including bacteria and viruses, multiplication and clinical disease, which results in large economic losses to the industry [29]. Studies of shrimp immune mechanisms could provide new strategies for disease prevention and control [30]. In the present study, we identified a FOXO in kuruma shrimp (*Marsupenaeus japonicus*). After knockdown *Foxo* expression, followed *Vibrio anguillarum* challenge, we found that the bacterial clearance ability and shrimp survival rate decreased significantly. Further study indicated that FOXO promoted the expression of AMPs directly or indirectly, and enhanced the phagocytosis of bacteria in the shrimp. The possible mechanisms of FOXO’s effects against pathogen infection in shrimp were analyzed.

## Results

### FOXO is expressed at basal levels in unchallenged shrimp and is upregulated in shrimp challenged with *V*. *anguillarum*

From hemocyte transcriptome sequencing of *M*. *japonicus*, we obtained the full-length cDNA sequence of *Foxo* (GenBank accession number: MW080526). Domain comparison showed that the predicted FOXO protein has a Forkhead (FH) domain, which is similar to *Homo sapiens* FOXOs including FOXO1, FOXO3, FOXO4, and FOXO6, and bears similar AKT phosphorylation (S1A Fig). The results of phylogenetic analysis showed that FOXOs were divided into two clusters including vertebrate and invertebrate FOXOs, and the shrimp FOXO was clustered in the invertebrate branch in the phylogenetic tree (S1B Fig). Similar to human FOXOs [31], shrimp FOXO has an extracellular regulated kinase (ERK) phosphorylation modification sites and CREB Binding Protein (CBP) acetylation modification sites, but has no sirtuin 1 (SIRT1) deacetylation modification sites and c-JUN N-terminal kinase (JNK) phosphorylation modification sites (S2 Fig).

**Fig 1:**
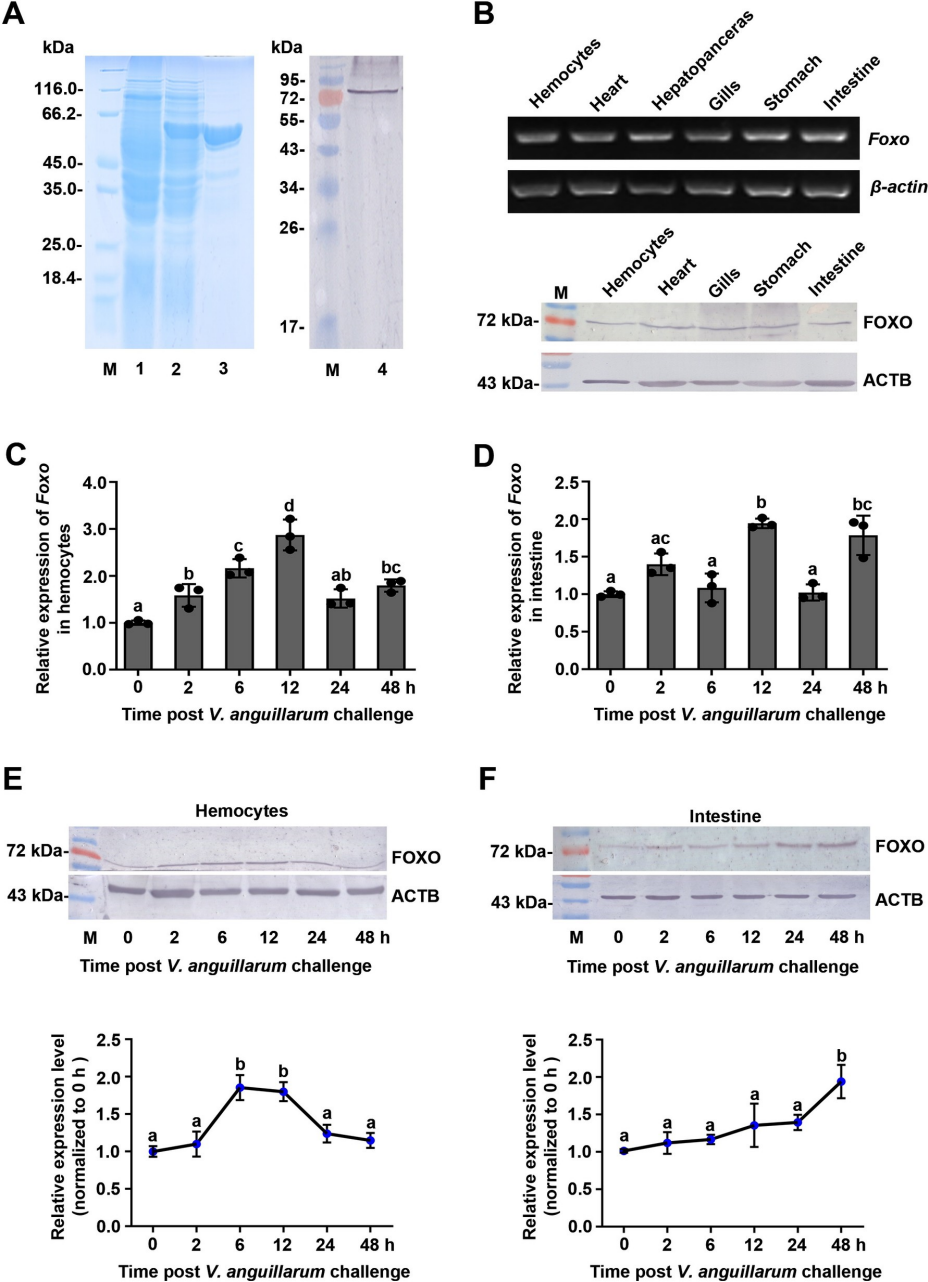
FOXO was upregulated in shrimp challenged by *V*. *anguillarum*. (**A**) Recombinant expression of the FH domain of FOXO in *E*. *coli* and western blotting to detect FOXO using polyclonal antibodies against FOXO prepared in rabbits. Lane 1, the total proteins of *E*. *coli* with *Foxo*-pGEX4T-1. Lane 2, the total proteins of the bacteria after IPTG induction. Lane 3, purified recombinant FOXO. Lane 4, FOXO in the intestines of untreated shrimp detected using western blotting. M, Protein molecular mass markers. **(B)** The tissue distribution of *Foxo* at mRNA (upper panel) and protein (lower panel) levels detected by RT-PCR and western blotting, respectively. ACTB is the abbreviation of β-actin. **(C, D)** The mRNA expression patterns of *Foxo* in hemocytes (C) and intestines (D) analyzed by qPCR using geometric mean expression of *β-actin* and *Ef-1α* as endogenous control genes. (**E, F)** The protein expression patterns of FOXO in hemocytes (E) and intestines (F) detected by western blotting. The results of statistical analysis of three replicates were shown in the lower panels. The bands on the western blot were digitalized using ImageJ software by scanning the bands from three independent repeats. Relative expression levels of FOXO/β-actin were expressed as the mean ± SD, and the value of the control shrimp was set as one. Error bars in the figure indicate SDs (three replicates).

Polyclonal antibodies recognizing shrimp FOXO were prepared using the recombinant FH domain of FOXO expressed in *Escherichia coli* (Fig 1A). Analysis of the tissue distribution of FOXO at the mRNA and protein levels revealed that it was expressed in all tested tissues (Fig 1B). The time course of FOXO expression in the hemocytes and intestines of shrimp challenged by *V*. *anguillarum* was analyzed using qPCR and western blotting. The results showed that FOXO was upregulated in the hemocytes and intestines at the mRNA level (Fig 1C and 1D) and at the protein level (Fig 1E and 1F). These results suggested that FOXO is involved in the response to *V*. *anguillarum* infection and prompted us to explore the function of FOXO in shrimp immunity.

### FOXO participates in homeostasis regulation of the hemolymph and intestinal microflora in healthy shrimp

**Fig 2:**
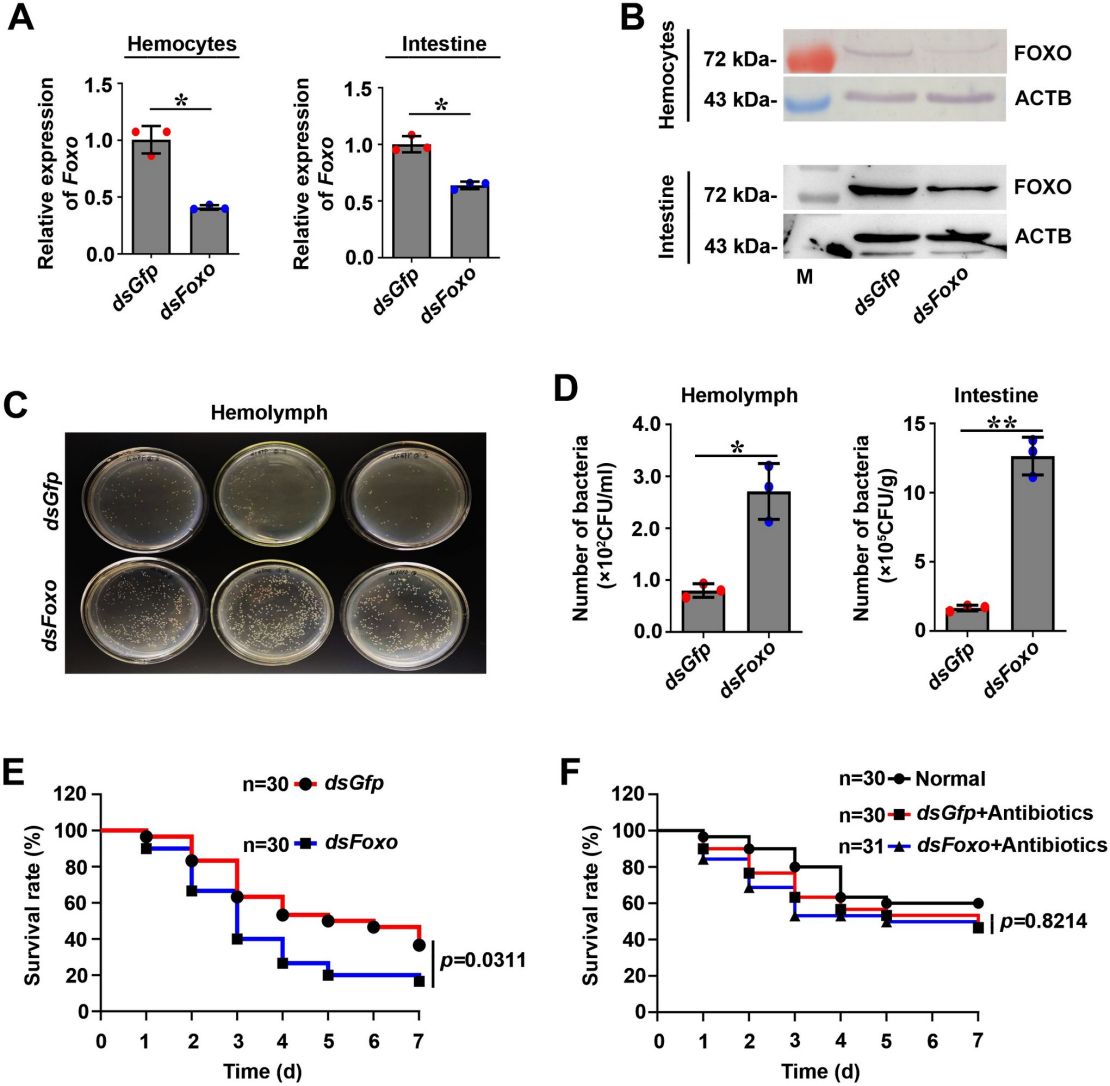
FOXO participates in the intestinal (local) and systemic antibacterial immune responses. **(A, B)** Efficiency of *Foxo*-RNAi in hemocytes (A) and intestines (B) of shrimp, as determined using qPCR (A) and western blotting (B). The qPCR date analysis using geometric mean expression of *β-actin* and *Ef-1α* as endogenous control genes. Error bars show SDs. **(C)** Detection of hemolymph bacteria of *Foxo-*knockdown shrimp by solid LB culture (three repeats); *dsGfp* injection was used as the control. The dilution ratio was 1:10. **(D).** The number of bacteria in the hemolymph and intestines in *Foxo*-knockdown shrimp and *dsGfp*-injection shrimp without bacterial challenge. **(E)**. The survival rate of *Foxo*-RNAi shrimp without bacterial challenge. *dsGfp* injection was used as the control. **(F)**. The survival rate of *Foxo*-RNAi shrimp treated with antibiotics. *dsGfp* injection was used as control. Normal: wild-type shrimp not treated with antibiotics.

To explore the function of FOXO in hemolymph and intestinal microflora homeostasis in shrimp, RNA interference was performed and the bacterial load was analyzed. The results showed that the expression of *Foxo* was decreased significantly in *Foxo*-RNAi shrimp (Fig 2A and 2B). Off-target effects of *Foxo* RNAi were also analyzed by detecting the expression of other *Fox* family genes (including *Foxk2* and *Foxn3*). The results showed that knockdown of *Foxo* did not decrease the expression levels of *Foxk2* or *Foxn3* (S3A–S3C Fig). Using western blotting and fluorescent immunocytochemistry assays, we further observed the subcellular distribution of FOXO in the intestines and hemocytes of shrimp challenged by bacteria after silencing of *Foxo*. The results showed that compared with the *dsGfp* group, the FOXO level in the nucleus decreased significantly in the *Foxo*-RNAi shrimp, even if the shrimp were infected by *V*. *anguillarum* (S3D–S4E’ Figs). All the above results suggested that our *Foxo* RNAi assay could specifically silence *Foxo* in hemocytes and intestines (including in the cytoplasm and nucleus). Next, we analyzed the bacterial load in the hemolymph and intestines after knockdown of *Foxo* in shrimp under normal conditions (without bacterial challenge). The results showed that the number of bacteria in the hemolymph and intestines were significantly increased after *Foxo* expression was successfully knocked down (Fig 2C and 2D). We further analyzed the survival rate of *Foxo*-RNAi shrimp without bacterial challenge, and the results showed that the survival rate of *Foxo*-knockdown shrimp decreased significantly compared with that of the control (Fig 2E). To further verify these results, we analyzed survival rate of *Foxo*-RNAi shrimp treated by antibiotics and the results showed that compared with the *dsGfp* group, the survival rate of the germ-free shrimp was not significantly decreased (Fig 2F). All the results suggested that FOXO plays a role in the homeostasis of the in vivo microbiota (including hemolymph and enteric microbiota) in shrimp under normal conditions.

### FOXO participates in the regulation of hemolymph and intestinal microflora homeostasis by promoting *Relish* and *Amp* expression in healthy shrimp

**Fig 3:**
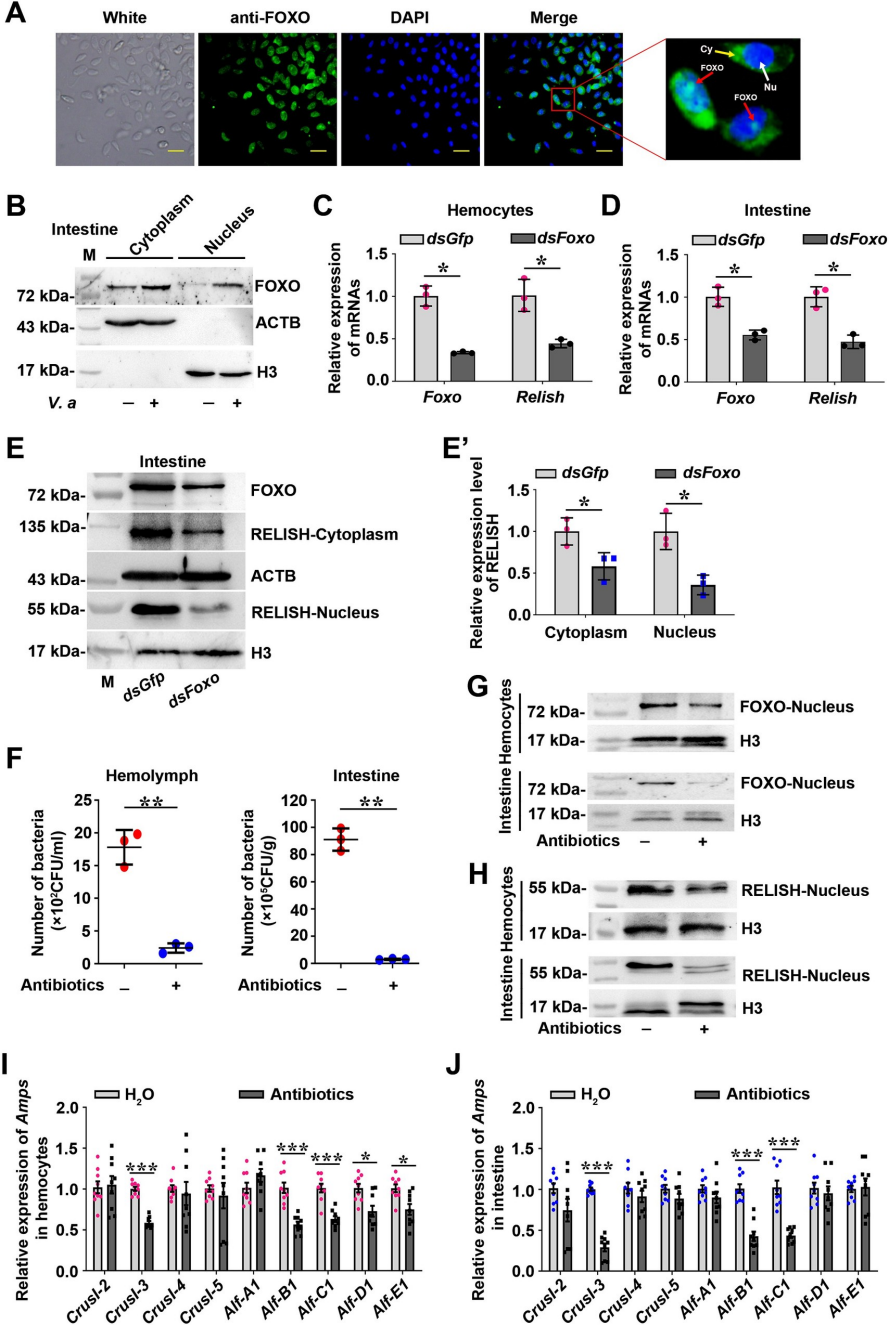
A basal amount of FOXO translocates into the nuclei of hemocytes and intestine cells and promotes *Relish* expression and subsequently activates the IMD pathway to regulate hemolymph and intestinal microbiota homeostasis under normal conditions. **(A)** Immunocytochemistry was used to detect the subcellular distribution of FOXO in hemocytes of normal shrimp. Scale bar = 20 μm. Cy: Cytoplasm; Nu: Nucleus. **(B)** Western blotting was used to detect the subcellular distribution of FOXO in intestine cells from normal shrimp and *V*. *anguillarum* infected shrimp. **(C)** The expression of *Relish* at mRNA level was detected in hemocytes of shrimp after knockdown of *Foxo* by qPCR using geometric mean expression of *β-actin* and *Ef-1α* as endogenous control genes. **(D)** The expression of *Relish* at the mRNA level in the intestines of shrimp after knockdown of *Foxo* was detected by qPCR using geometric mean expression of *β-actin* and *Ef-1α* as endogenous control genes. **(E)** The RELISH level was detected using western blotting in shrimp after knockdown of *Foxo*. **(E’)** Three replicates of panel E were digitalized using ImageJ software. **(F)** The bacterial load of hemolymph and intestines in shrimp 24 h post antibiotics treatment. **(G)** FOXO in nucleus was detected using western blotting in hemocytes and intestines of shrimp 24 h post treatment with antibiotics. **(H)** RELISH in nucleus was detected using western blotting in shrimp hemocytes and intestines at 24 h post treatment with antibiotics. **(I, J)** The expression of *Amps* in hemocytes (I) and intestines (J) of germ-free shrimp determined by qPCR using *β-actin* and *Ef-1α* as an internal reference gene. Normal shrimp (H_2_O injection) was used as the control. The data were analyzed statistically using the Mann-Whitney U test. All the error bars in the figure indicate SDs.

Previous studies demonstrate that healthy shrimp contain low but stable numbers of bacteria in the circulating hemolymph, and high albeit constant amounts of bacteria in gastrointestinal tract, and the immune effectors, such as antimicrobial peptides (AMPs) regulated by the nuclear factor kappa B (NF-κB) pathway and reactive oxygen species (ROS) regulated by the dual oxidase (DUOX) pathway, play an essential role in homeostasis hemolymph and intestinal microbiota [32–34]. To explore the mechanism of FOXO’s involvement in the regulation of hemolymph and intestinal microflora homeostasis under normal conditions, we first detected the subcellular distribution of FOXO in hemocytes and intestines under normal condition. The results showed a weak signal of FOXO in the nuclei of shrimp hemocytes and intestine cells under normal conditions (Fig 3A and 3B), suggesting that a little FOXO was activated by the *in vivo* microbiota in shrimp under normal conditions. AMP expression is regulated by the IMD pathway in the intestines of *Drosophila* [35]. Therefore, we assumed that the transcription factor *Relish* might be a target gene of FOXO. We detected the mRNA expression of *Relish*, the transcription factor of the IMD pathway, in shrimp hemocytes and intestines after knockdown of *Foxo*. The results showed that *Relish* expression decreased significantly in *Foxo*-knockdown shrimp (Fig 3C and 3D), and similar results were obtained at the protein level (Fig 3E and 3E’). The results suggested that FOXO could promote the expression of *Relish* and activate the IMD pathway. To confirm that FOXO participates in hemolymph and gastrointestinal tract homeostasis through IMD signaling, germ-free shrimp were obtained (Fig 3F) and RELISH activation and AMP expression were detected. Compared with that in the control, the amount of FOXO and RELISH in the nuclei of intestinal cells and hemocytes decreased significantly (Fig 3G and 3H). Meanwhile, the expression of AMPs, including Crustins (*CrusI-2* to *5*) and Anti-lipopolysaccharide factors (*Alf A1*, *B1*, *C1*, *D1* and *E1*), possibly regulated by FOXO and/or RELISH was screened in the germ-free shrimp, and results showed that expression of *CrusI-3*, *Alf-B1* and *Alf-C1* decreased significantly in hemocytes and intestines of the shrimp (Fig 3I and 3J). Given that the expression of *Alf-B1* and *Alf-C1* was regulated by IMD pathway [36], these results suggested that FOXO participates in the regulation of homeostasis of the hemolymph and gastrointestinal tract under normal conditions through the IMD pathway to promote the expression of AMPs.

### FOXO is activated in shrimp by *V*. *anguillarum* challenge and participates in the pathogen clearance of the hemolymph and intestines

**Fig 4:**
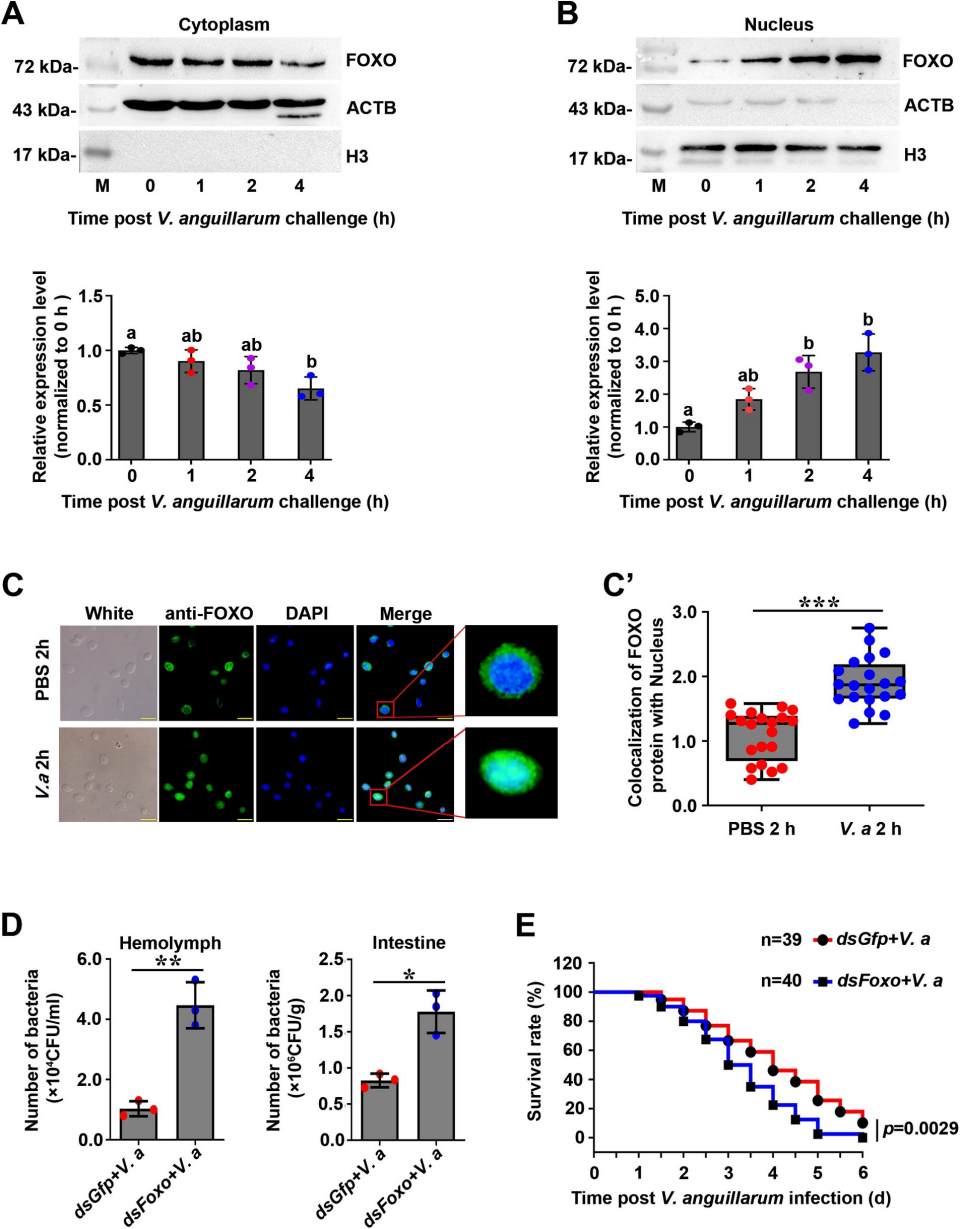
FOXO translocated into nucleus in shrimp challenged by *V*. *anguillarum*. **(A, B)** The FOXO distribution patterns in the cytoplasm (A) and nucleus (B) of intestine cells of shrimp challenged by bacteria, as analyzed using western blotting (lower panels of A and B). The western blotting bands were digitalized using ImageJ software by scanning the bands from three repeats. The relative expression levels of FOXO/β-actin or FOXO/Histone 3 were expressed as the mean ± SD, and the value of the control shrimp was set as one. **(C)** The nuclear translocation of FOXO in hemocytes of shrimp 2 h post *V*. *anguillarum* challenge was detected using fluorescent immunocytochemical assays. PBS injection was used as control. Scale bar = 20 μm. **(C’)** Statistic analysis of panel C. the colocalization of FOXO and DAPI-stained nuclei in hemocytes was analyzed by WCIF ImageJ software. **(D)** The bacterial number in the hemolymph and intestines of *Foxo*-knockdown shrimp followed *V*. *anguillarum* injection; *dsGfp*-injection in shrimp following bacterial injection was used as control. **(E)** The survival rate of *Foxo*-RNAi shrimp infected by *V*. *anguillarum*. *dsGfp* injection was used as the control.

It is reported that FOXO can be activated by bacterial or cytokine stimulation and is translocated to the nucleus to regulate the expression of certain target genes in mammals [17]. To detect whether FOXO was activated in shrimp challenged by bacteria, the subcellular distribution of FOXO in intestines of the shrimp was analyzed using western blotting. The results showed that the amount of FOXO in the cytoplasm of intestinal cells decreased gradually from 2 to 4 h post *V*. *anguillarum* challenge (Fig 4A). In contrast, the amount of FOXO in the nucleus of intestine cells increased significantly after infection of *V*. *anguillarum* (Fig 4B). To analyze the role of FOXO in systemic immunity, a fluorescent immunocytochemical assay was used to observe the nuclear translocation of FOXO in hemocytes of shrimp 2 h post *V*. *anguillarum* stimulation. We found that bacterial challenge-induced FOXO translocation into nucleus increased significantly but there was no obvious change in PBS challenged shrimp (Fig 4C and 4C’). To explore whether the nuclear translocated FOXO is involved in pathogen clearance, we analyzed the bacterial load in the hemolymph and intestines in *Foxo*-knockdown shrimp at 6 h after bacterial challenge. We found that after knockdown of *Foxo*, the bacteria load in the hemolymph and intestines increased significantly compared with that in the control groups (Fig 4D). Moreover, the survival rate of *Foxo*-knockdown shrimp infected with *V*. *anguillarum* decreased significantly compared with that of the control (Fig 4E). These results indicated that FOXO was activated in enteric cells and hemocytes, and suggested that FOXO participates local and systemic immune responses against bacterial infection.

### Pathogen infection decreases the activity of AKT and induces FOXO nuclear translocation

**Fig 5:**
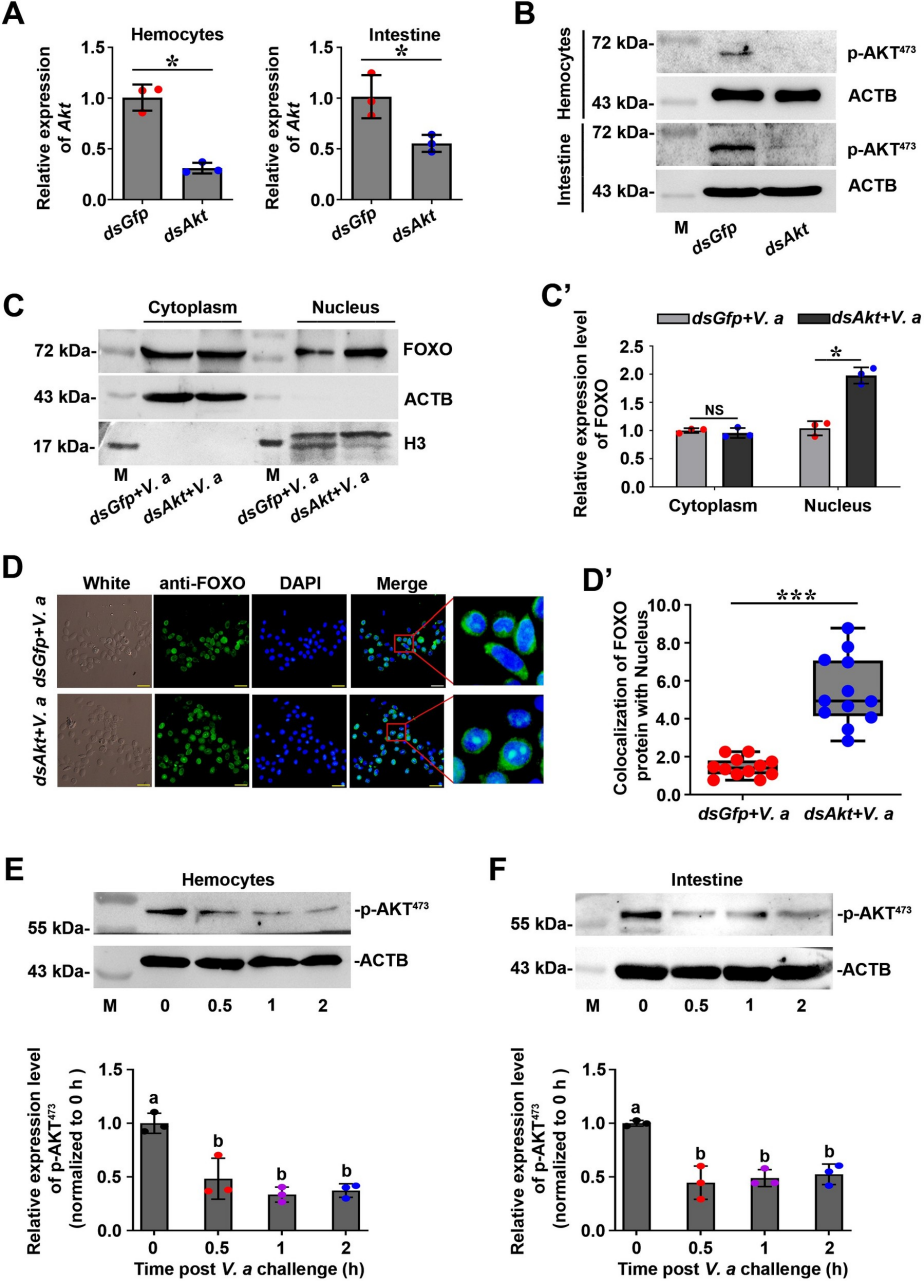
Pathogen infection decreased AKT activity, which promoted FOXO nuclear translocation in hemocytes and enteric cells of shrimp. **(A, B)** Efficiency of *Akt*-RNAi in the hemocytes and intestines of shrimp, as determined by qPCR using geometric mean expression of *β-actin* and *Ef-1α* as endogenous control genes (A) and western blotting (B). **(C)** The amount of FOXO in the cytoplasm and nucleus of intestines from *Akt*-knockdown shrimp analyzed by western blotting. *dsGfp* was used as control. **(C’)** The statistical analysis of panel (C). ImageJ software was used to digitalized the bands by scanning three-repeat pictures. **(D)** The nuclear translocation of FOXO in hemocytes of *Akt*-knockdown shrimp was detected 2 h post *V*. *anguillarum* challenge using a fluorescent immunocytochemical assay. *dsGfp* was used as control. Scale bar = 20 μm. **(D’)** Statistical analysis of (D). WCIF ImageJ software was used to analyze the co-localization by fluorescence intensity ratio of anti-FOXO (green) and DAPI-stained nucleus (blue) in hemocytes after fixed the exposure value. **(E, F)** Content change of phosphorylated AKT (p-AKT) in hemocytes (E) and intestines (F) of shrimp challenged by *V*. *anguillarum*. The lower panels are digitalized figures of (E) and (F) based on bands of western blotting and statistical analysis, respectively.

Previous studies indicated that FOXO transcriptional activity is regulated by phosphorylation modification and that the serine/threonine kinase AKT regulates the activity of Forkhead transcription factors by phosphorylating them and inhibiting their unclear translocation [9,37]. To investigate whether the increase of FOXO in the nucleus after pathogenic bacteria infection is affected by AKT, we first detected the content of FOXO in the cytoplasm and nucleus by western blotting after knockdown of *Akt* of shrimp. The results showed that the amount of FOXO in the nucleus of hemocytes and intestinal cells increased significantly after knockdown of *Akt* expression (Fig 5A and 5B) in shrimp 2 h post infection with *V*. *anguillarum* (Fig 5C and 5C’). To verify the experimental results, we also used an immunocytochemistry assay to observe the subcellular distribution of FOXO in hemocytes of shrimp after knockdown of *Akt*. The results showed that FOXO was significantly increased in the nucleus of hemocytes when *Akt* was knocked down 2 h post infection with *V*. *anguillarum* (Fig 5D and 5D’). To explore whether the pathogen infection affects AKT activity, we detected the phosphorylation change of AKT after pathogen infection using a human p-AKT (Ser^473^) antibody (Phosphorylation site of shrimp AKT is Ser^486^), and found that the level of phosphorylated AKT decreased significantly in hemocytes and intestines of shrimp after 30 min of *V*. *anguillarum* infection (Fig 5E and 5F). These results suggested that pathogen infection can reduce the level of the phosphorylated AKT, which inhibits the enzyme activity, and decreases the ability of AKT to phosphorylate FOXO. The non-phosphorylated FOXO enters the nucleus and induces the expression of its target genes.

### FOXO exerts immune defense against invading bacteria by regulating the expression of antimicrobial peptides in shrimp infected by bacteria

**Fig 6:**
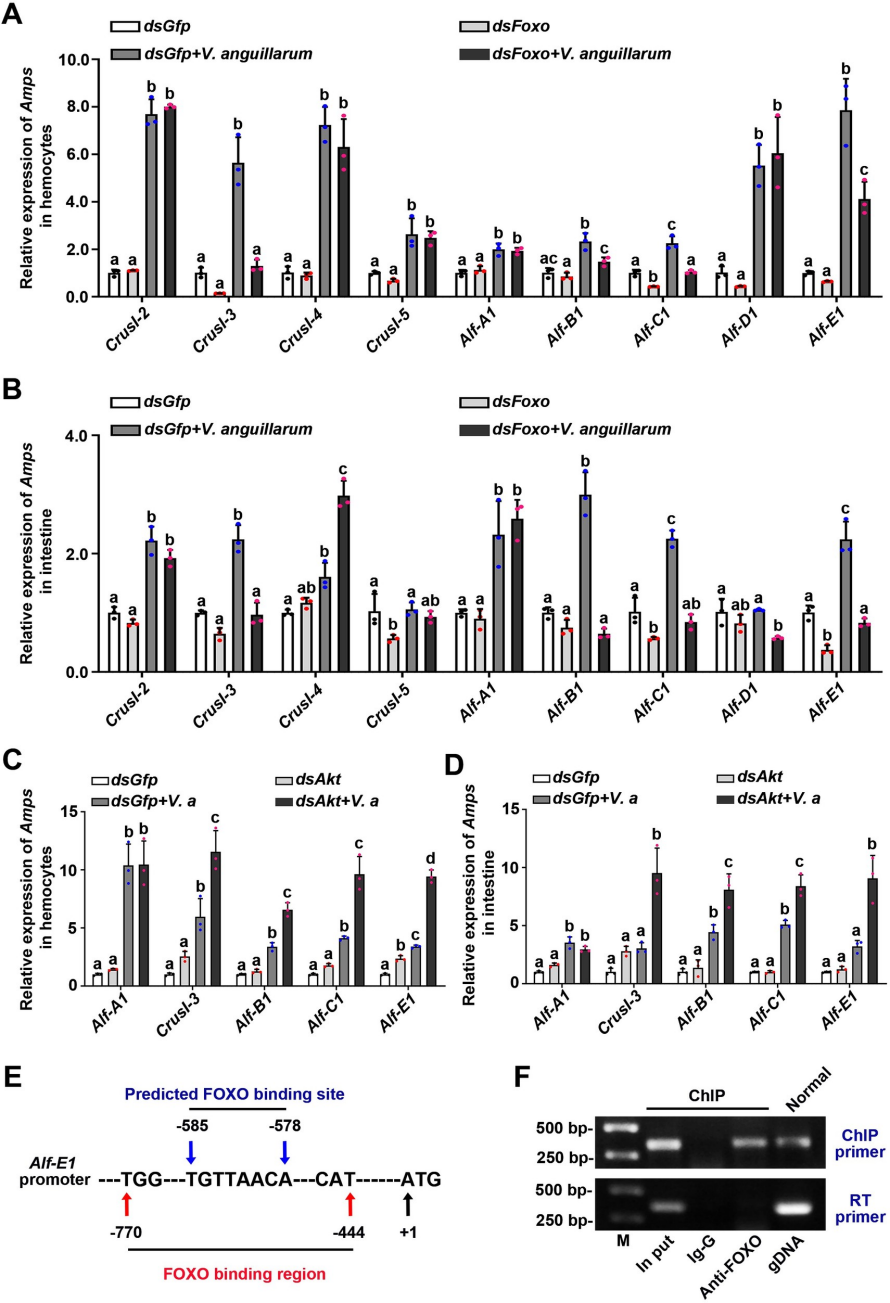
FOXO performs its antibacterial roles in shrimp by regulating the expression of AMPs. **(A, B)** The expression of *Amps* in hemocytes (A) and intestines (B) of *Foxo*-knockdown shrimp, as determined by qPCR using geometric mean expression of *β-actin* and *Ef-1α* as endogenous control genes; *dsGfp* injection in shrimp was used as the control. The data were analyzed statistically using the Mann-Whiteny U test. **(C, D)** The expression of *Amps* in hemocytes (C) and intestines (D) of *Akt*-knockdown shrimp was analyzed by qPCR using geometric mean expression of *β-actin* and *Ef-1α* as endogenous control genes; *dsGfp* injection in shrimp was used as the control. Significant differences were analyzed using one-way ANOVA. **(E)** Schematic diagram of the predicted binding sites of FOXO on the *Alf-E1* promoter. **(F)** ChIP and RT-PCR were used to detect FOXO binding to the *Alf-E1* promoter sequence using primers (*Alf-E1*-F and *Alf-E1*-R, Table 1). RT-PCR was also used to amplify encoding region of *Alf-E1* with RT-PCR primers (*Alf-E1*-RT-F and *Alf-E1*-RT-R. Table 1) using the ChIP obtained DNA as template. IgG antibody was used as control, and the normal genomic DNA amplified fragment was used to confirm the target band.

To explore the mechanism by which FOXO affects the response against bacterial infection in shrimp, we first identified the immune effector genes regulated by FOXO. After the successful knockdown of *Foxo* in hemocytes and intestines of shrimp with or without *V*. *anguillarum* challenge, the expression levels of *CrusI-3*, *Alf-B1*, *Alf-C1* and *Alf-E1* decreased significantly in the silenced shrimp compared with those in the control group (Fig 6A and 6B). To further verify the results, we also detected the expression of four AMPs after knockdown of *Akt*. The results showed that the expression of the four AMPs increased significantly 6 h post *V*. *anguillarum* infection compared with that in the control group (Fig 6C and 6D). Compared with upregulated AMPs (including *Alf-B1*, and *Alf-C1*) under normal condition (Fig 3I and 3J), additional gene of AMPs (*CrusI-3*, *Alf-E1*) was also upregulated in the shrimp infected by *V*. *anguillarum* (Fig 6A and 6B). Our previous study indicated that RELISH promoted the expression of *Alf-B1* and *Alf-C1* in the shrimp [36]. These results indicate that FOXO might directly regulate AMP expression, such as *CrusI-3* and *Alf-E1*, independent of the IMD signaling pathway. To confirm the hypothesis, a ChIP assay was performed to detect if FOXO could bind to the promoter of *Alf-E1*. The promoter sequence of *Alf-E1* was obtained (S4 Fig) and FOXO binding sites was predicted (Fig 6E). The ChIP result showed that FOXO could bind to the *Alf-E1* promoter (Fig 6F). These results suggested that FOXO also participates in antibacterial immunity by directly regulating the expression of AMPs in shrimp.

### FOXO promotes phagocytosis of hemocytes against bacteria by promoting SRC expression

**Fig 7:**
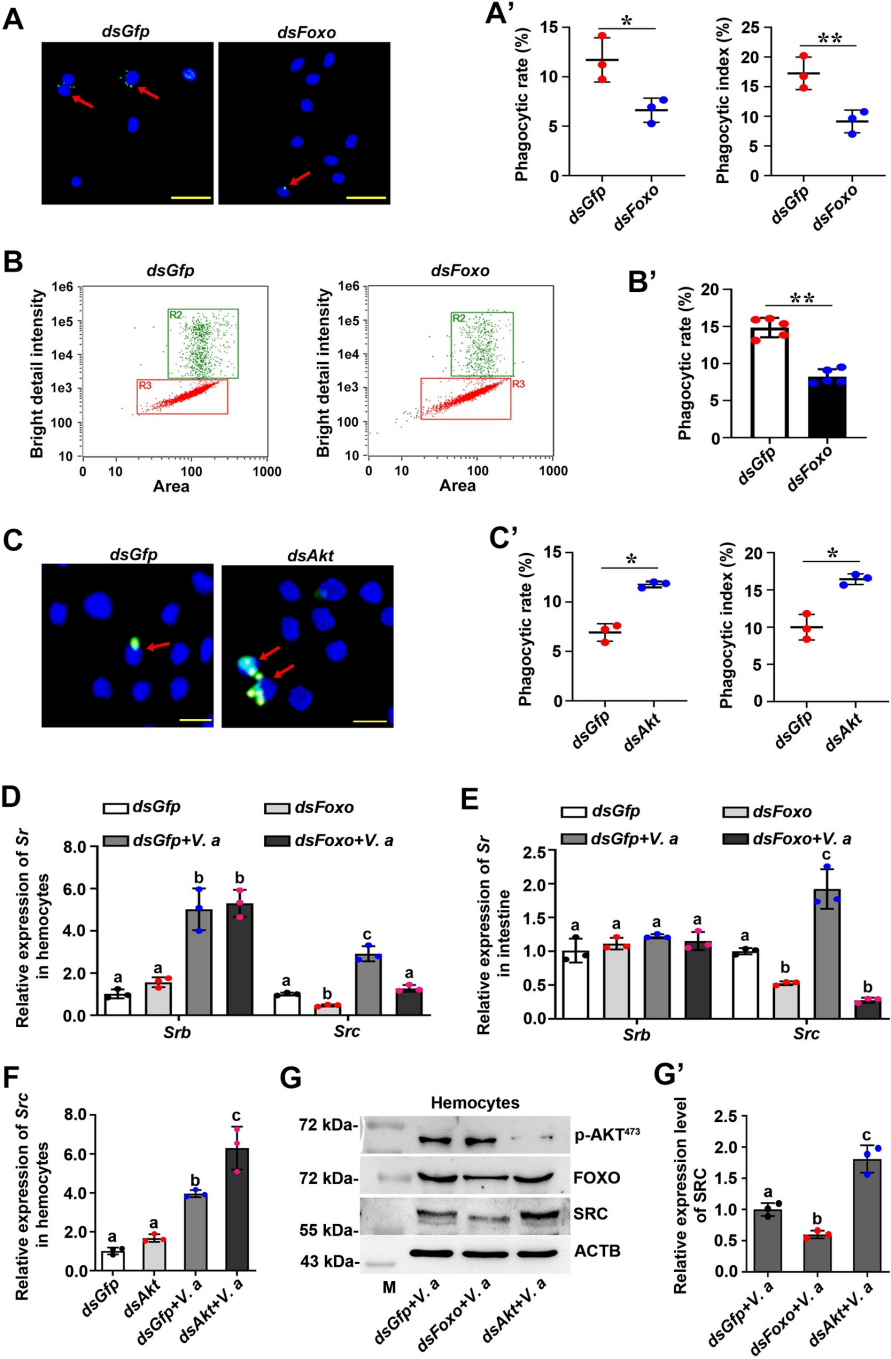
FOXO enhances hemocyte phagocytosis of pathogens in shrimp by promoting SRC expression. **(A)** Hemocyte phagocytosis of *V*. *anguillarum* observed using a fluorescent immunocytochemical assay under a fluorescence microscope. *V*. *anguillarum* was labeled with FITC (green) and cell nuclei were stained with DAPI (blue). The red arrow indicates the phagocytic hemocytes. Scale bar = 20 μm. **(A’)** Statistical analysis of the phagocytic rate and phagocytic index in *Foxo*-knockdown shrimp. The *dsGfp* injection group was used as the control. Five hundred hemocytes were counted under the fluorescence microscope in each experiment. The data were analyzed statistically using Student’s *t*-test. **(B)** Bacterial phagocytosis by hemocytes analyzed using flow cytometry. The phagocytosed hemocytes containing bacteria (R2) were separated from non-phagocytosed hemocytes (R3) based on fluorescence signal of labeled bacteria in hemocytes using IDEAS Application v6.0 software. **(B’)** The phagocytic rate of hemocytes based on the flow cytometry data. Three thousand hemocytes were counted for each analysis and three repetitions were carried out. The *dsGfp* group was used as the control. **(C)** Hemocyte phagocytosis of *V*. *anguillarum* in *Akt*-knockdown shrimp observe under the fluorescence microscope. Scale bar = 10 μm. **(C’)** Statistical analysis of phagocytic rate and phagocytic index based on data in panel (C). **(D)** The mRNA expression level of *Srb* and *Src* in hemocytes from *Foxo*-knockdown shrimp analyzed by qPCR using geometric mean expression of *β-actin* and *Ef-1α* as endogenous control genes. *dsGfp* injection was used as the control. **(E)** The mRNA expression of *Srb* and *Src* in intestines of *Foxo*-RNAi shrimp analyzed by qPCR using geometric mean expression of *β-actin* and *Ef-1α* as endogenous control genes. **(F)** The mRNA expression of *Src* in hemocytes of *Akt*-RNAi shrimp analyzed by qPCR using geometric mean expression of *β-actin* and *Ef-1α* as endogenous control genes. **(G)** The protein level of *SRC* in hemocytes of *Foxo*-RNAi or *Akt*-RNAi shrimp, as analyzed using western blotting. *dsGfp*-injection used as control. **(G’)** The statistical analysis based on data of three replicates of panel (G).

Previous studies have reported that FOXO interacts directly with the promoter regions of *CXCR2* and *CD11b* to regulate the phagocytosis of neutrophils in mammals [26]. To test whether FOXO is associated with hemocyte phagocytosis in shrimp antibacterial immunity, we examined the phagocytic rate after interference of *Foxo* using different methods. After knockdown of *Foxo*, the phagocytic rate and phagocytic index of the *Foxo-*knockdown group were significantly lower than those of the control group (*dsGfp*) (Fig 7A and 7A’). We also used flow cytometry to quantify the phagocytic rate of hemocytes after silencing of *Foxo*. The results showed that the phagocytosis rate of the *Foxo*-RNAi group also decreased significantly compared with that of the *dsGfp* group (Fig 7B and 7B’). To further validate these results, we also examined the phagocytic rate after knockdown of *Akt*. The results showed that compared with those of the control group, the phagocytic rate and phagocytic index increased significantly (Fig 7C and 7C’). These results suggested that FOXO promotes phagocytosis of hemocytes in shrimp.

To explore how FOXO enhances the hemocyte phagocytosis in shrimp, we detected the expression of previously reported genes involved in phagocytosis of shrimp. Scavenger receptor class B (SRB) and class C (SRC) are reported to be involved in hemocyte phagocytosis [38,39]. We found that the mRNA expression level of *Src*, rather than *Srb*, decreased significantly after silencing of *Foxo* in the hemocytes and intestines (Fig 7D and 7E), whereas the expression of *Src* increased significantly after knockdown of *Akt* in shrimp (Fig 7F). Similar results were obtained at the protein level (Fig 7G and 7G’). These results suggested that FOXO promotes hemocyte phagocytosis of pathogens in shrimp by promoting SRC expression.

### FOXO promotes the expression of RAB5 and RAB7 during phagocytosis in shrimp

Previous studies have shown that the small GTPases, RAB5 and RAB7, are the key regulators of the transport of newly endocytosed vesicles to early and late endosomes [40]. RAB5 is a key regulator of the initial fusion events and is a marker of early endosomes [41,42]. RAB7 is needed for the late phagosome-lysosome fusion and is a marker of late endosomes [43]. To explore whether RAB5 and RAB7 are involved in pathogen clearance in shrimp, we firstly analyzed the expression patterns of *Rab5* and *Rab7*, and found that the two small GTPases were upregulated in shrimp challenged with bacteria (S5A–S5D Fig). We further analyzed the survival rate of *Rab5*-RNAi and *Rab7*-RNAi shrimp after bacterial challenge, and the results showed that the survival rate of *Rab5* and *Rab7* knockdown shrimp decreased significantly compared with that of the control (S5E and S5F Fig). These results suggested that RAB5 and RAB7 play important functions in resistance against bacterial pathogens.

**Fig 8:**
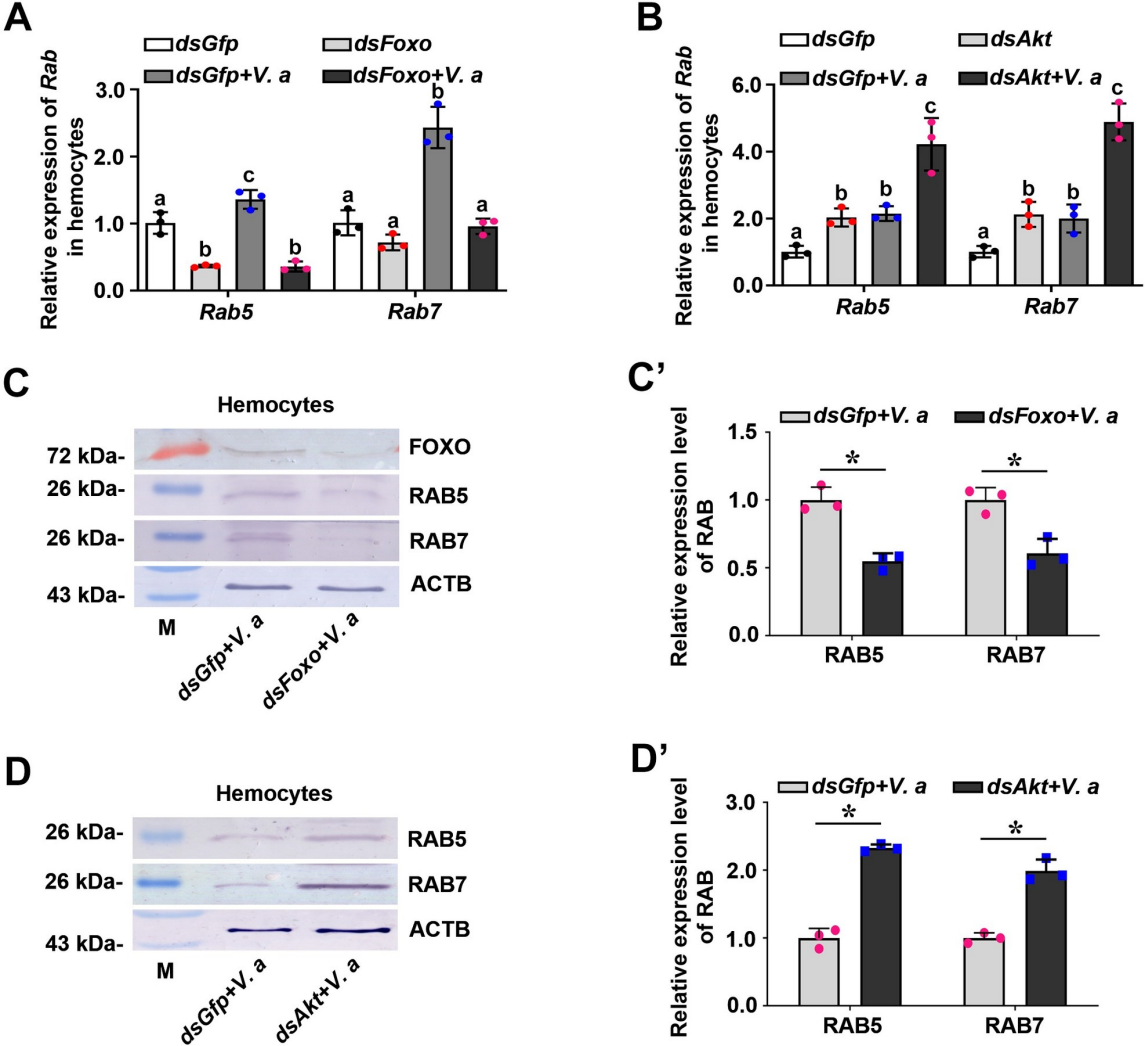
RAB5 and RAB7 are involved in FOXO-mediated phagocytosis of hemocytes in shrimp. **(A, B)** qPCR was used to analyze the mRNA expression levels of *Rab5* and *Rab7* in the hemocytes of *Foxo*-knockdown shrimp (A) and *Akt*-knockdown shrimp (B) with and without bacterial infection. The qPCR used geometric mean expression of *β-actin* and *Ef-1α* as endogenous control genes. **(C)** The protein levels of the RAB5 and RAB7 in hemocytes of *Foxo*-knockdown shrimp at 2 h post *V*. *anguillarum* challenge determined using western blotting. (**C’**) The statistical analysis based on data of three replicates of panel (C). **(D)** The protein levels of the RAB5 and RAB7 in hemocytes of *Akt*-knockdown shrimp at 2 h post *V*. *anguillarum* challenge determined using western blotting. (**D’**) The statistical analysis based on data of three replicates of panel (D).

**Fig 9:**
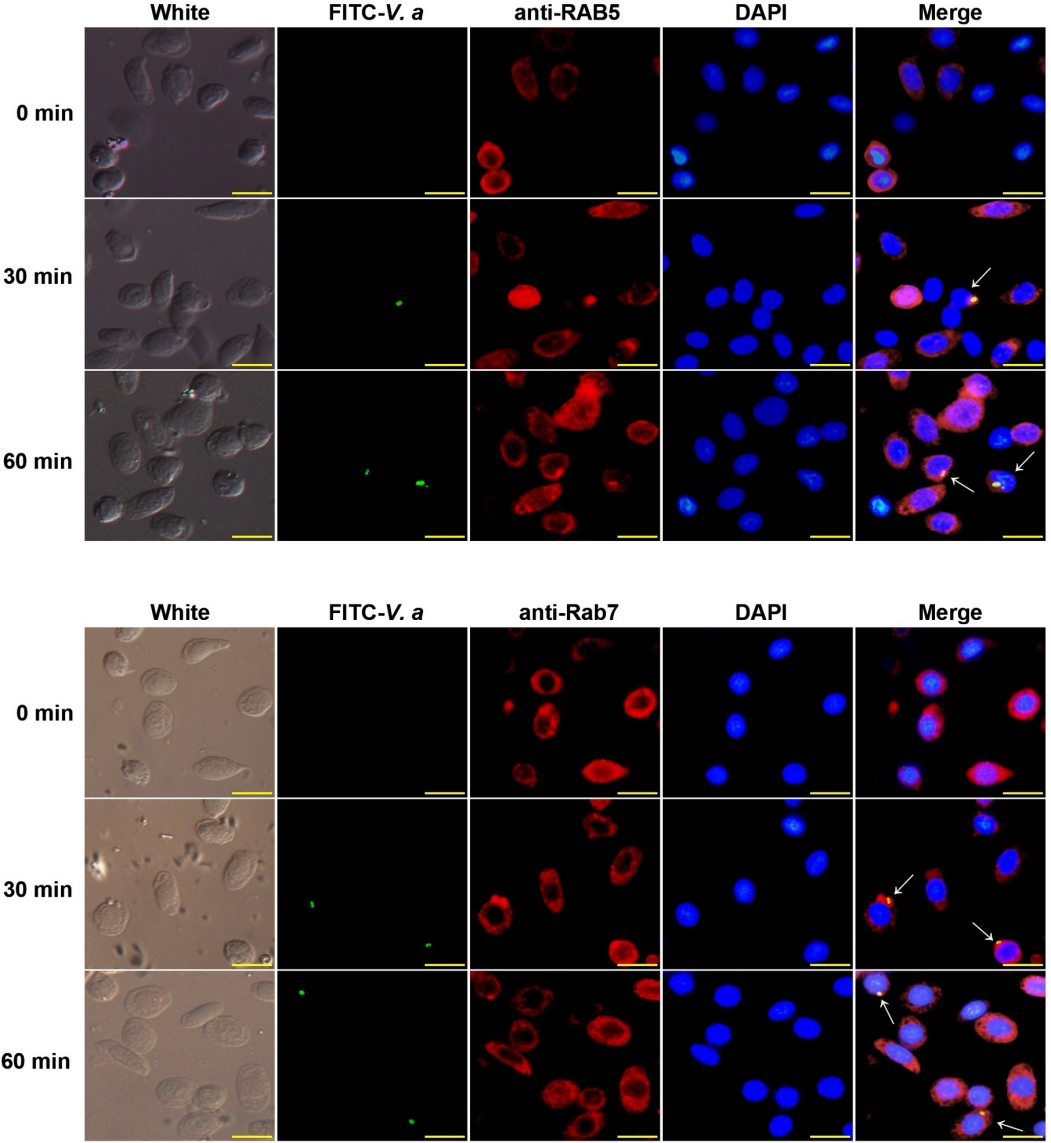
RAB5 and RAB7 co-localize with *V*. *anguillarum*. The co-localization of RAB5 and RAB7 with FITC labeled-*V*. *anguillarum* in shrimp hemocytes, as analyzed using a fluorescent immunocytochemical assay. The bacteria were labeled with FITC (green) and injected into shrimp. Hemocytes were collected from five shrimp at 30 min and 1 h after bacterial injection and incubated with anti-RAB5 or anti-RAB7 antibodies. The secondary antibody was antirabbit IgG Alexa-546 (red). Nuclei were stained with DAPI (blue). Co-localization in hemocytes is indicated by white arrows. Scale bar = 10 μm.

To explore if RAB5 and RAB7 are involved in FOXO-mediated phagocytosis, we examined the mRNA expression levels of *Rab5* and *Rab7* after interference with *Foxo* expression in shrimp. The results showed that the expression levels of *Rab5* and *Rab7* decreased significantly in the hemocytes and intestines (Figs 8A and S6A). whereas the expression of *Rab5* and *Rab7* increased significantly after knockdown of *Akt* in shrimp (Fig 8B). To further confirm the above results, the protein levels of RAB5 and RAB7 in *Foxo* or *Akt* knockdown shrimp were also detected. Compared with the *dsGfp* injection group, RAB5 and RAB7 levels also decreased significantly in the hemocytes and intestines in *Foxo*-knockdown shrimp but increased significantly in *Akt*-knockdown shrimp at 2 h post stimulation by *V*. *anguillarum* (Figs 8C–8D’; S6B and S6B’). These results suggested that RAB5 and RAB7 participate in FOXO-mediated phagocytosis of hemocytes in shrimp. We further detected that RAB5 and RAB7 were co-localized with *V*. *anguillarum* in shrimp hemocytes (Fig 9). Taken together, the results suggested that RAB5 and RAB7 are involved in FOXO-mediated phagocytosis in shrimp.

## Discussion

**Fig 10:**
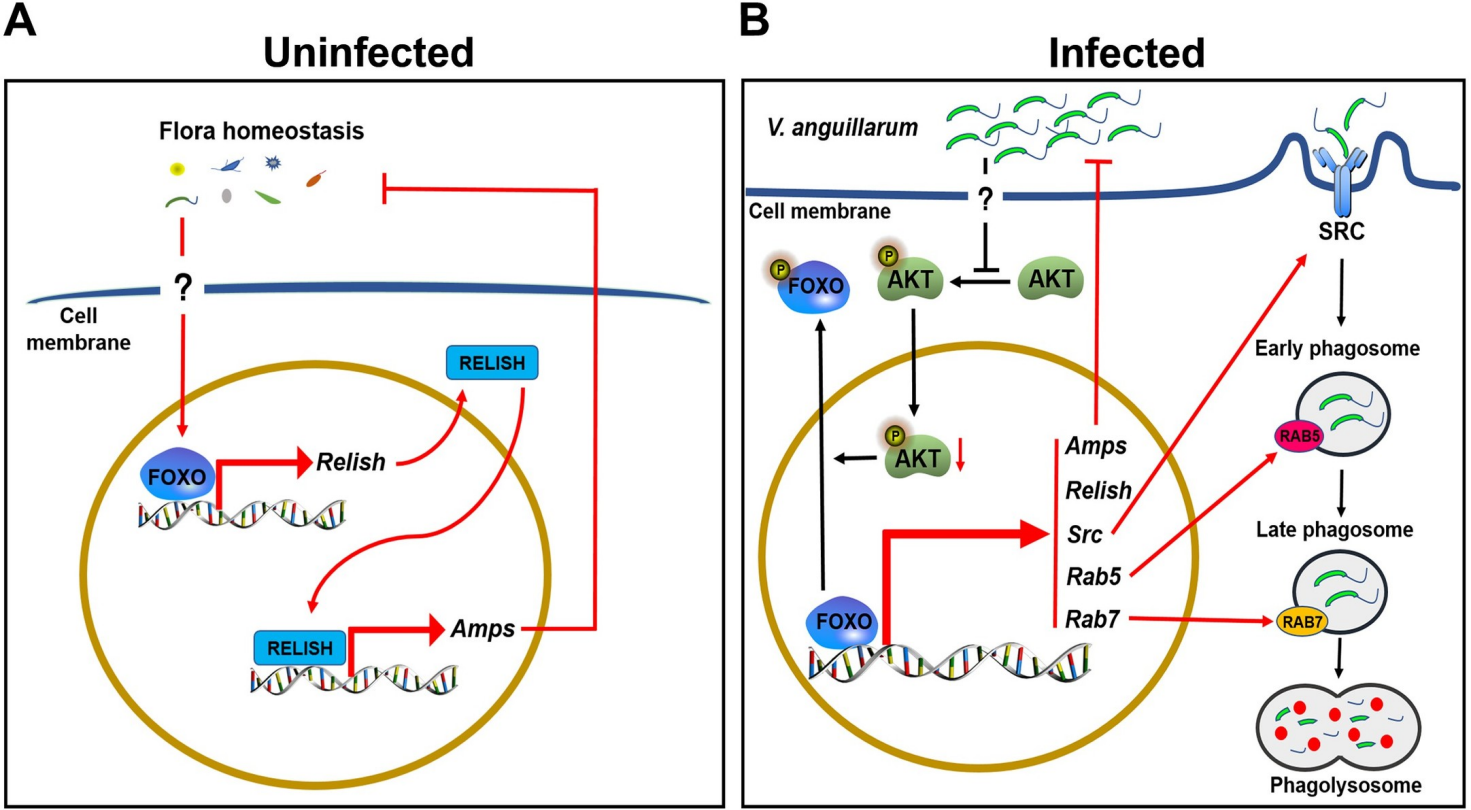
Schematic representation of FOXO involvement in local and systemic innate immunity in shrimp. **(A)** In uninfected conditions, FOXO maintains the homeostasis of the hemolymph and the intestinal microbiota by promoting *Relish* and subsequently AMP expression. **(B)** In infected conditions, AKT phosphorylation is decreased by pathogen infection, resulting in increasing of FOXO content in the nucleus. FOXO directly promotes the expression of Relish and AMPs to eliminate invasive pathogens; on the other hand, FOXO promotes the phagocytosis of pathogens through the SRC-mediated phagocytosis pathway.

FOXO, as a very important transcription factor, participates in many physiological and metabolic functions [44,45]. In the present study, we identified a FOXO from shrimp (*M*. *japonicus*) and found that it participates in the regulation of homeostasis of the hemolymph and enteric microbiota by regulating AMP expression under normal conditions. FOXO can be activated by bacterial infection in shrimp and translocated into the nucleus, where it regulates directly and indirectly (by IMD pathway) the expression of AMPs. FOXO upregulated expression of phagocytosis-related genes, such as SRC and small GTPases, RAB5 and RAB7, to promote hemocyte phagocytosis against pathogens. Therefore, shrimp FOXO plays different roles in systemic and local (enteric) immune responses against pathogens (Fig 10).

Compared with human FOXOs, shrimp FOXO has a relatively conserved amino acid sequence and AKT phosphorylation sites; however, the second AKT phosphorylation site of shrimp FOXO is threonine instead of serine. AKT phosphorylation of FOXO controls the nuclear and cytoplasmic transport of FOXO [9]. In this study, we found that AKT is related to the control of FOXO’s nuclear entry. After *V*. *anguillarum* infection, the level of phosphorylated AKT was significantly reduced, which indicated that AKT activity was decreased in pathogen-infected shrimp, thus increasing the nuclear retention or nuclear translocation of FOXO, and subsequently inducing the expression of target genes, such as *Relish* and *Amp*, to resist against pathogen infection.

Previous studies have found that FOXO participates in the homeostasis of *Drosophila* intestinal microflora by repressing the expression of peptidoglycan recognition protein SC2 (the negative regulator of IMD pathway) to active the pathway for AMP expression [14]. RELISH was reported as a key target gene of FOXO in the control of hypoxia tolerance in *Drosophila* [46]. Like other animals, shrimp gastrointestinal tract contains relatively high and constant microbiota. Increasing evidence also reveals that the hemolymph of some aquatic healthy invertebrates, including shrimp also harbors stable amounts of bacteria [47]. Our previous study found that a C-type lectin participates in homeostasis regulation of hemolymph microbiota by maintaining the expression of AMPs [34], but we do not know what signal pathways involved in the regulation of AMP expression. Here, our results suggested that FOXO is involved in the regulation of hemolymph and enteric microflora homeostasis through IMD signaling under normal conditions. In uninfected shrimp, knockdown of *Foxo* resulted in significantly reduced expression of *Relish*, and the shrimp survival rate decreased significantly, even without bacterial infection. We also found a small amount of FOXO in the nucleus of normal shrimp cells. Therefore, we think that FOXO participates in the homeostasis regulation of hemolymph and enteric flora under normal conditions by promoting *Relish* expression and thereafter enhancing AMP expression by the IMD pathway. These results suggested that FOXO participates in local and systemic immunity to promote microbiota homeostasis of the hemolymph and gastrointestinal tract in shrimp.

Humoral immunity plays a critical role in the resistance to pathogen infection in invertebrates via the production of a battery of AMPs [21,48]. The humoral response comprises the inducible synthesis and secretion of AMPs, which are released into the hemolymph as a systemic response, or released onto the epidermal surface or the cavity of gastrointestinal tract as a local response [19]. AMP expression is mainly regulated by the Toll/Dif and IMD/Relish signaling pathways in fruit fly [49]. Currently, several families of AMPs have been identified in shrimp, including crustins, anti-lipopolysaccharide factors (Alfs), and penaeidins [50]. The present study showed that FOXO can directly or indirectly (through IMD/Relish signaling) regulate the expression of AMPs, including *CrusI-3*, *Alf-B1*, *Alf-C1*, and *Alf-E1*. We thought that low activation of FOXO in the hemocytes and intestines of shrimp under normal conditions could regulate the key target gene of *Foxo*, i.e., *Relish*, to maintain the homeostasis of the *in vivo* microbiota. During infection, the invading pathogens activate FOXO, most of which is translocated into the nucleus, where it regulates more target genes, including some AMPs, such as *Alf-E1* and *CrusI-3*, in addition to *Relish*. Taken together, FOXO participates in the regulation of hemolymph and intestinal microbiota homeostasis in healthy shrimp via basal level activation of IMD signaling in the hemolymph and gastrointestinal tract. Pathogen infection activates FOXO by decreasing AKT activity and promotes the expression of AMPs directly and indirectly in systemic immunity and local (intestinal) immunity.

Phagocytosis is a receptor-mediated, actin and ATP-dependent, phenomenon that is triggered by the binding of particles or pathogens to specific cytomembrane receptors, and represents an important immune process through which the host can protect itself from pathogens [24]. Small GTPases, such as RAB5 and RAB7, are involved in continued membrane remodeling and vesicle traffic in phagocytosis. Our previous study reported that scavenger receptors SRB and SRC are involved in hemocyte phagocytosis as receptors in shrimp [38,39]. In the present study, we found that after knockdown of *Foxo*, *Src* expression decreased markedly, and the hemocyte phagocytosis rate decreased significantly, suggesting that *Src* is a target gene of FOXO, and that FOXO is involved in SRC-mediated phagocytosis of pathogenic bacteria. FOXO could also promote the expression of two small GTPase, RAB5 and RAB7. These small GTPases are involved in the sequential transport of, and are the key determinants of, early and late phagosomes [51]. Phagosomes migrate from the periphery of the cell to the intracellular region, with RAB5 being replaced by RAB7 during transport, and the final goods are transported to lysosome for degradation [52,53]. We also found that RAB5 and RAB7 could co-localize with the pathogens in hemocytes, which indicated that the pathogens were phagocytized by hemocytes in shrimp by passing through early and late phagosomes, before finally being degraded in lysosomes. Therefore, FOXO is involved in the cellular immune response by promoting phagocytosis of hemocytes.

In conclusion, our results indicate that FOXO not only participates in microbiota homeostasis regulation under normal conditions, but also in the clearance of pathogenic bacteria in infected shrimp by promoting the expression of AMPs and phagocytosis of hemocytes.

## Materials and methods

### Ethics statement

All animal experiments were performed in compliance with China National Directives (GB 14922.2–2011 and GB 14925–2010). The rabbit experiments for antibody preparation in the study were carried out in accordance with protocols approved by the Animal Care & Welfare Committee at Shandong University School of Life Sciences (SYDWLL-2021-54). The approval according to the regulations on the use of shrimp in our study was not necessary because our research used a limited number of common shrimp frequently consumed by the wider community.

### Animals

Healthy shrimp (*Marsupenaeus japonicus*), weighing about 6–10 g each, were obtained from the aquatic product market in Qingdao, Shandong province, China. Before the experiment, shrimp were cultured for at least 1 day in the shrimp culture system with aerated seawater at 23–25°C for acclimation to the new environment.

### Bioinformatic analysis

The full-length cDNA sequence of *Foxo* was obtained from hemocyte and intestinal transcriptome sequencing of *M*. *japonicus* (BGI, Shenzhen, China). The open reading frame (ORF) of *Foxo* was amplified using a pair of primers (*Foxo*-EX-F and *Foxo*-EX-R; Table 1) for sequence confirmation. The corresponding cDNA was conceptually translated to obtain the deduced protein sequence using the ExPASy-Translation tool (http://cn.expasy.org/). Similarity analysis was carried out using BLAST (http://blast.ncbi.nlm.nih.gov/Blast.cgi/), and the domain architecture prediction was performed using SMART (http://smart.emblheidelberg.de). A phylogenetic tree of FOXOs from different species was constructed using the MEGA 6.0 program [54].

**Table 1:** Sequences of primers used in this study.

**Primers**	**Sequence (5’-3’)**	**GenBank number**	**T_m_^1^ (°C)**	**Size^2^ (bp)**	**Efficiency (%)^3^**
RT-PCR and qPCR					
*Foxo*-F	TTGCACCTTGATTTCCGTAG	MW080526	52.5	217	104.64
*Foxo*-R	CACTTGTGGAGTCTTTCCGTAG		55.3		
*Foxk2*-F	TACTTCGAGACGCCCCACTT	MW080527	58.6	214	92.44
*Foxk2*-R	TTTTGCGTTTGGGAGGAGA		54.2		
*Foxn3*-F	CAGTGACTACGGCGAGGAAT	MW080528	57.1	238	94.54
*Foxn3*-R	GTAGGGGAAGTGGTCAAGGA		56.4		
*Akt-*F	GTTGACTGGTGGGGTTATGGA	KP419289	57.1	243	106.06
*Akt*-R	GGTGATGTAGAAGGGGTGATT		54.0		
*Srb*-F	TGCCCACCTCACAAACTCAC	KP121407	58.1	130	93.69
*Srb*-R	GCACACAACGCATCACACTT		56.6		
*Src*-F	TCTCCCACAGAGGCTACTTC	KU213605	56.1	189	99.39
*Src*-R	CGCTTCGGTCGTTTGATT		53.2		
*Rab5*-F	ATTTGAGATTTGGGACACGG	MW419290	52.6	244	99.66
*Rab5*-R	ATAGGTCTGGGCCTCTTCAT		55.1		
*Rab7*-F	ATCGCGGAGCTGATTGTT	MW419291	54.4	192	96.52
*Rab7*-R	ACTGTTGTGCTCGCTTCGTC		58.4		
*β-actin*-F	AGTAGCCGCCCTGGTTGTAGAC	GU645235	55.3	240	107.78
*β-actin*-R	TTCTCCATGTCGTCCCAGT		54.6		
*Ef-1α-*F	GGATTGCCACACCGCTCACA	AB458256	61.4	223	98.83
*Ef-1α-*R	CACAGCCACCGTTTGCTTCAT		58.9		
*Relish-F*	AGGATGAAGATGAGGAGGAA	MN607236	53.3	241	104.27
*Relish-R*	GAGATGTCAATGCCCGAGT		55.0		
*CrusI-2*-F	GCGTTTTCGTCTTCGTCCTG	MT977626	56.9	102	91.96
*CrusI-2*-R	AGTCCTTTCCGCCGTCACA		59.6		
*CrusI-3*-F	CTCCACCACTCTCGCACTAACA	MT977627	59.2	205	97.47
*CrusI-3*-R	TGATGGTCTCAGATTGGGGC		57.4		
*CrusI-4*-F	TACTGTTGGCAGCCGTGTCT	MT977628	59.4	175	96.92
*CrusI-4*-R	GGTTGAATCTGGGTTTGAGGA		54.7		
*CrusI-5*-F	ATCGGCAAACCCGCAGTCTCTCT	KU213606	63.5	172	94.58
*CrusI-5*-R	CCGCTCTTCGTCGCAGCAGTAATAGT		63.1		
*Alf-A1*-F	CTGGTCGGTTTCCTGGTGGC	KU213607	62.2	219	102.11
*Alf-A1*-R	CCAACCTGGGCACCACATACTG		61.3		
*Alf-B1*-F	CGGTGGTGGCCCTGGTGGCACTCTTCG	KY627759	72.8	244	104.17
*Alf-B1*-R	GACTGGCTGCGTGTGCTGGCTTCCCCTC		72.3		
*Alf-C1*-F	CGCTTCAAGGGTCGGATGTG	KU213608	59.3	149	107.4
*Alf-C1*-R	CGAGCCTCTTCCTCCGTGATG		60.7		
*Alf-D1*-F	CTTTGGCGTGGAACAAGGTAGAGGAT	KU160499	61.1	243	92.84
*Alf-D1*-R	GCTTTTTATTTTGGGGGTCACGCTGT		60.3		
*Alf-E1*-F	TCCTAACCACGCAGTGCTTTGCTAATG	KY627760	61.4	216	94.8
*Alf -E1*-R	GCTTTTCGGATTTGCCTTCGATGTTTG		59.2		
**Primers**	**Sequence (5’-3’)**				
Recombinant expression					
*Foxo*-EX-F	TACTCAGAATTCATGATGGCAACCAGTTTC				
*Foxo* -EX-R	TACTCACTCGAGTTATGACATCTTCATGCAGTC				
ChIP					
*Alf-E1*-F	TGGAATAAACCTGTAATGTTCAA				
*Alf-E1*-R	AATGCTTATTGTCGCTG				
RNAi					
*Foxo*-Ri-F	GCGTAATACGACTCACTATAGGATGTGAACGGAACAGTGAGGC				
*Foxo*-Ri-R	GCGTAATACGACTCACTATAGGCAGACAACAAGCGGGGATT				
*Rab5*-Ri-F	GCGTAATACGACTCACTATAGGTATTATCGTGGTGCTCAGGC				
*Rab5*-Ri-R	GCGTAATACGACTCACTATAGGGTCACTCTTTGGCAGTTTCT				
*Rab7*-Ri-F	GCGTAATACGACTCACTATAGGCATCTCCCAACACCTTCA				
*Rab7*-Ri-R	GCGTAATACGACTCACTATAGGGATACCGCCCTATTCTCC				
*Gfp*-Ri-F	GCGTAATACGACTCACTATAGGTGGTCCCAATTCTCGTGGAAC				
*Gfp*-Ri-R	GCGTAATACGACTCACTATAGGCTTGAAGTTGACCTTGATGCC				
*Akt*-Ri-F	GCGTAATACGACTCACTATAGGGGGCGAGAAGCTCAAAGAA				
*Akt*-Ri-R	GCGTAATACGACTCACTATAGGAACCCCACCAGTCAACACCT				

1 Annealing temperature (°C)

2 Amplicon size (bp)

3 qPCR assay efficiencies of related genes (%)

### Bacterial challenge and sample collection

*Vibrio anguillarum* (ATCC 43305) obtained from Marine College, Shandong University (Weihai, China) and now preserved in our laboratory. *V*. *anguillarum* was cultured overnight at 37°C in LB medium and re-cultured for 4 hours at 1:100 inoculation on the second day. The bacteria in logarithmic growth phase were collected by centrifugation at 5000 × g for 5 min at 4°C and washed two times with phosphate-buffered saline (PBS: 10 mM Na_2_HPO_4_, 140 mM NaCl, 2.7 mM KCl, and 1.8 mM KH_2_PO_4_, pH 7.4). *V*. *anguillarum* resuspended in phosphate-buffered saline for shrimp challenge. Approximately 2 ×10^7^ colony forming units (CFU)/shrimp were injected into the abdomen of each shrimp using a microsyringe. The control group was injected with same volume of PBS. The hemocytes, heart, hepatopancreas, gill, stomach, and intestines were collected from at least three shrimp for RNA and protein extraction. For hemocyte collection, the hemolymph was extracted from the ventral sinus using a syringe preloaded with 0.8 ml of anticoagulant buffer (10% sodium citrate), and then quickly centrifuged at 700 × *g* for 8 min at 4°C for hemocyte isolation.

### RNA and protein extraction, and cDNA synthesis

Total RNA was extracted from the hemocytes and different organs of shrimp using the TRIzol (ET101, Transgen, Beijing, China). Proteins from the different organs were extracted using Radioimmunoprecipitation assay (RIPA) Lysis Buffer (P0013B, Beyotime, Jiangsu, China). First strand cDNA synthesis was performed using a cDNA Synthesis Kit (5x All-in-One RT MasterMix; Applied Biological Materials-abm, Vancouver, Canada), following the manufacturer’s instructions.

### Recombinant expression and antibody preparation

The cDNA sequence of *Foxo* was amplified by reverse transcription polymerase chain reaction (RT-PCR) using primers *Foxo*-EX-F and *Foxo*-EX-R (Table 1). The purified PCR products were then digested using restriction enzymes and ligated into plasmid pGEX-4T-1 (GE Healthcare, Piscataway, NJ, USA). and the constructed plasmids were transformed into *Escherichia coli* Rosseta (DE3) cells. Protein expression was induced using 1 mM isopropyl-β^-^D-thiogalactopyranoside at 37°C. The recombinant proteins were subjected to affinity chromatography using glutathione (GST) resin (C600031, BBI, Shanghai, China), following the manufacturer’s instructions. Rabbit antisera against FOXO was prepared according to a previously described method [55].

### Western blotting

The proteins extracted from different organs or hemocytes were separated using 10% sodium dodecyl sulfate polyacrylamide gel electrophoresis (SDS-PAGE). The proteins in the SDS-PAGE gels were transferred to a cellulose nitrate membrane using transfer buffer (25 mM Tris–HCl, 20 mM glycine, 0.037% mM SDS, and 20% ethanol) and incubated in 5% skim milk or 3% bovine serum albumin (BSA) in Tris-buffered saline (TBS: 150 mM NaCl, 10 mM Tris-HCl, pH 8.0) for 1 h with gentle shaking at room temperature. The membranes were then incubated with primary antiserum: Anti-FOXO at a 1:100 dilution, anti-RAB5 at 1:25, anti-Rab7 at 1:25, anti-beta actin (ACTB) at 1:250 (prepared in our laboratory); Histone-3 polyclonal antibodies (A2348, ABclonal, Wuhan, China, 1:2500); anti-phosphorylated (p)-AKT polyclonal antibodies (WLP001a, Wanleibio, Shenyang, China, 1:500) and shaken gently overnight at 4°C. After washing three times with TBST (0.1% Tween-20 added to TBS), the membranes were incubated with secondary antibody (ZB2308 ZSGB-Bio, Beijing, China, 1:5,000) or (ZB2301 ZSGB-Bio, Beijing, China, 1:5,000) for 3 h at room temperature with gentle shaking. The immunoreactive protein bands were developed using a nitrotetrazolium blue chloride (A610379, BBI) and P-toluidine salt (A610072, BBI) solution under dark conditions or using enhanced chemiluminescence (ECL). β-actin or Histone-3 were used references.

### Tissue distribution and expression patterns in shrimp challenged by bacteria

The tissue distribution of *Foxo*/FOXO in the hemocytes, heart, gill, sputum, stomach, and intestines were analyzed using RT-PCR with primers *Foxo*-F and *Foxo*-R (Table 1) and by western blotting with anti-FOXO antibodies, respectively. The PCR procedure consisted of an initial incubation at 94°C for 3 min; followed by 35 cycles of 94°C for 30 s, 50°C for 30 s, and 72°C for 30 s; followed by 72°C for 10 min. The PCR products were analyzed using agarose gel electrophoresis (1.5% agarose). The β-actin gene (*β-actin*-F and *β-actin*-R; Table 1) was used as the internal control.

The time course expression patterns of *Foxo/*FOXO at the RNA and protein levels in shrimp challenged with *V*. *angu*illarum were analyzed using quantitative real-time PCR (qPCR) in a thermal cycler (qTOWER3, ANALYTIK JENA AG, Jena, Germany) and by western blotting, respectively. The qPCR procedure comprised: 95°C for 10 min; 40 cycles at 95°C for 10 s and 60°C for 50 s; and then a melting period from 65°C to 95°C. The expression profiles of *Foxo* were produced using the 2^-ΔΔCT^ method [56] using the geometric mean of the two internal control genes of *β-actin* and *Ef-1α* for normalization. The efficiency of primer pair in qPCR was analyzed following the MIQE method [57] with a 10-fold logarithmic dilution of a cDNA mixture to generate a linear standard curve.

### Isolation of nuclear and cytoplasmic proteins

The intestine tissue was dissected from shrimp and washed in PBS three times. Protein extraction was carried out by using a nuclear and cytoplasmic protein extraction kit (R0050, Solarbio, Beijing, China) following the manufacturer’s instructions. At least three shrimp were used for protein extraction to eliminate individual differences.

### RNA interference assay

To analyze gene function, we performed RNA interference (RNAi) assays. Several pairs of primers containing the T7 promoter sequence (*Foxo*-RI-F and *Foxo*-RI-R; *Rab5*-RI-F and *Rab5* -RI-R; *Rab7*-RI-F and *Rab7* -RI-R; *Akt*-RI-F and *Akt*-RI-R. Table 1) were designed to amplify the templates for dsRNA synthesis. Using the T7 RNA polymerase (EP0111, Thermo Fisher Scientific, Waltham, MA, USA) and cDNA template, we synthesized the dsRNA fragments. Double-strand Green fluorescent protein *dsGfp* RNA served as the control, which was amplified using *Gfp*-RI-F and *Gfp* -RI-R (Table 1) as primers for *dsGfp* synthesis. Then, the *dsRNA* fragments (50 μg/each) were injected into the abdominal segment of *M*. *japonicus* using a microsyringe and a second injection was carried out 12 h later using the same dose. RNA interference efficiency was analyzed using quantitative real-time reverse transcription PCR (qRT-PCR, comprising reverse transcription of RNA to form cDNA, which was then used as the template in a qPCR assay) or western blotting 24 h post second injection. *β-actin* was used as the internal reference.

### Bacterial clearance and survival rate assays

After knockdown of the genes of interest, bacterial clearance assays were performed. First, the shrimp were challenged with *V*. *anguillarum* (2 × 10^7^ CFU), collected at 6 h post-injection, washed thoroughly with sterile PBS and 75% ethanol, and carefully dissected to remove the entire intestines of the shrimp, which were placed in a sterilized 1.5 ml EP tube for weight determination. The intestines were homogenized in sterile PBS for bacterial number detection. Hemolymph was also collected using the above-mentioned method and the plasma was obtained by centrifugation. The intestinal homogenate and plasma sample were diluted, and then dropped and spread on solid Luria-Bertani (LB) medium plates overnight at 37°C, and the number of bacteria was counted.

For analysis of the survival rate, shrimp were divided into two groups (n ≥ 30 shrimp per each group). After 24 h of *dsRNA* injection, the shrimp were injected with bacteria *V*. *anguillarum* (2 × 10^6^ CFU) and the dead shrimp were monitored every 12 h after bacterial injection. *dsGfp* injection was used as the control. For analysis of the survival rate of germ-free shrimp, the *Foxo*-RNAi shrimp were treated by antibiotics (see the following method), dead shrimp were monitored every 12 h. The survival rate of each group was calculated and the survival curves were analyzed statistically using Kaplan–Meier plots.

### Antibiotic treatments

To analyze whether a low activation of FOXO could induce IMD signaling in normal animals, germ-free animals were obtained and used to analyze expression of AMPs following a previously reported method [34]. Ampicillin and kanamycin were added to the seawater to a final concentration of 25 mg/liter to generate an axenic environment. Each shrimp was injected with ampicillin and kanamycin (25 μg each/animal) or fed orally with ampicillin and kanamycin (2.5 mg each/animal) to generate axenic animals. The same dose of sterile water was used as control. After 24 h, RNA and proteins of shrimp were extracted respectively and the bacterial load in the shrimp was analyzed using previously reported method [34].

### Chromatin immunoprecipitation (ChIP)

To detect the target genes of FOXO, a ChIP assay was performed. Firstly, the promoter binding site prediction website (http://jaspardev.genereg.net/) was used to predict the possible promoter binding site of FOXO. Six hours after normal shrimp were infected by *V*. *anguillarum* (1 × 10^7^ CFU*)*, the hemocytes of five shrimp were collected and ChIP analysis was carried out using a ChIP kit (P2078, Beyotime) according to the manufacturer’s instructions. A pair of primers were designed according to the promoter sequence of the *Alf-E1* promoter (*Alf-E1*-F and *Alf-E1*-R; Table 1), and the target band was amplified using RT-PCR. The PCR products were analyzed using agarose gel electrophoresis (1.5% agarose). RT-PCR primers (*Alf-E1*-F and *Alf-E1*-R, Table 1) were used as a reference to exclude the influence of genomic DNA and cDNA.

### Bacterial phagocytosis assay

The bacteria in logarithmic growth phase were collected by centrifugation at 5000 × g for 5 min at 4°C and washed twice with PBS. Fluorescent isothiocyanate (FITC) (Sigma, St. Louis, MO, USA) was used to label *V*. *anguillarum* using a previously reported method [39]. The FITC-labeled bacteria (1 × 10^7^) were injected intramuscularly into shrimp. Then, the hemolymph was drawn at 2 h post injection using a sterile 5 ml syringe preloaded with 0.5 ml of anticoagulant buffer and 0.5 ml 4% paraformaldehyde. After washing twice with sterile PBS by centrifugation, the hemocytes were stained using 4′,6-diamidino-2-phenylindole (DAPI) for 10 minutes and dropped to polylysine coated slides for observation under fluorescence microscopy (Olympus BX51, Tokyo, Japan). The phagocytosis rate was defined as [the number of hemocytes with ingested bacteria/ the number of all cells observed or tested] × 100%. The phagocytotic index was defined as [the number of total bacteria/ the number of all cells observed or tested] × 100%. To confirm the above results, the phagocytosis rate of hemocytes were also assayed using flow cytometry (Becton Dickinson, Franklin Lakes, NJ, USA). The FITC-labeled bacteria (1 × 10^7^) were injected intramuscularly into shrimp. Then, the hemocytes of 5 shrimp were collected at 2 h post injection and washing twice with sterile PBS by centrifugation. No staining was performed for the whole hemocytes in the phagocytotic analysis, other than the labeling bacteria with FITC. Before flow cytometry analysis, the whole system of flow cytometry was calibrated and the gates (cell size) was set in flow cytometry to remove impurities and hemocyte debris. Afterwards the hemocytes were collected by flow cytometry (Becton Dickinson, Franklin Lakes, NJ, USA) and used the fluorescence detection channel (488 nm) to detect FITC fluorescence signal. About 3000 hemocytes were collected in each group. The fluorescence intensity of the collected cells was screened and the phagocytosed hemocytes containing bacteria (R2) were separated from non-phagocytosed hemocytes (R3) by fluorescence signal. Each group of flow cytometry analysis contained 3000 hemocytes and 5 repetitions were performed. The phagocytosis rate was analyzed by IDEAS Application v6.0 software.

### Fluorescent immunocytochemical assay

The hemolymph was extracted from the ventral sinus of shrimp, and hemocytes were collected using the above-mentioned method. The collected hemocytes were washed three times with PBS and were dropped on polylysine coated slides and incubated for 3 h. After treatment with 0.2% Triton X-100, the hemocytes were washed with PBS three times, and then blocked with 3% BSA (PBS dilution) at 37°C for 1 h. The hemocytes on the slide were incubated with 3% BSA-diluted antibody (1:50 dilution) and incubated overnight at 4°C. The next day, the hemocytes were incubated with 3% BSA-diluted goat anti-rabbit Alexa 488 or antirabbit IgG Alexa-546 (diluted at 1:500) for 1 h, after washed five times with PBS. Then, the hemocytes were washed five times with PBS and stained using DAPI for 10 minutes. Finally, after three washes with PBS, the slide was observed under a fluorescence microscope (Olympus BX51, Japan). WCIF ImageJ software (NIH, Bethesda, MD, USA) was used to analyze the colocalization of FOXO and DAPI-stained nuclei in the hemocytes.

### Co-location of RAB5 or RAB7 with *V*. *anguillarum*

After FITC staining, *V*. *anguillarum* (3 × 10^7^ CFU) was injected into shrimp, and hemocytes were collected using the above-mentioned method for a fluorescent immunocytochemistry experiment. The co-localization of bacteria with RAB5 or RAB7 was observed under a fluorescence microscope (Olympus BX51, Japan) and analyzed using WCIF ImageJ software [58].

### Statistical analysis

Data were presented as the mean ± standard deviation (SD) of at least three replicates. Significance differences were analyzed using Student’s *t*-test for paired comparisons or one-way ANOVA for multiple comparisons. Asterisks in figures indicate statistical significance (*p < 0.05, **p < 0.01, and ***p < 0.001). The different lowercase letters indicate significant differences (*p* < 0.05) in the ANOVA analysis. The nonparametric test, Mann-Whitney U test was also used for the un-normally distributed data. The survival rate was calculated and the survival curves are presented as Kaplan–Meier plots and the statistically using a log-rank test. All statistical analysis were produced using GraphPad 8.0 data view software. Western blotting bands were analyzed based on three independent replicates using ImageJ software (National Institutes of Health, http//imagej.nih.gov/ij/download.html).

## Supporting information

S1 Fig Bioinformatic analysis of FOXOs from shrimp and other species. **(A)** Comparison of the FOXO domain architectures and AKT/PKB phosphorylation sites between *M*. *japonicus* and *Homo sapiens*. The FOXO amino acid sequence of *Homo sapiens* were obtained from GenBank (FOXO1, GenBank accession number: AAH70065.3; FOXO3, AAH68552.1; FOXO4, AAI06762.1; FOXO6, ARQ84049.1). **(B)** Phylogenetic tree of FOXOs from different species. The FOXO sequences of different species were obtained from GenBank, and the NJ tree was established using MEGA 6.0. The results were repeated 1000 times by bootstrapping. FOXO of *M*. *japonicus* is marked by a red triangle.
(TIF)

S2 Fig Sequence alignment of FOXOs from human and shrimp using GeneDoc software. The posttranslational modification sites of FOXOs are shown in the alignment following previous reports on FOXOs. NLS: nuclear localization signal; NES: nuclear export sequence. CBP: cyclic-AMP responsive element binding (CREB)-binding protein; JNK: c-JUN N-terminal kinase; ERK: extracellular regulated protein kinase; SIRT1: Sirtuin1.
(TIF)

S3 Fig Detection of the efficiency and off-target effects of *Foxo* RNAi in shrimp. **(A)** Efficiency of *Foxo*-RNAi in hemocytes and intestines analyzed using qPCR. **(B)** mRNA expression of *Foxk2* in *Foxo*-RNAi shrimp detected using qPCR. **(C)** mRNA expression of *Foxn3* in *Foxo*-RNAi shrimp analyzed using qPCR. **(D)** Western blotting analysis of FOXO protein in the cytoplasm and nuclei of intestine cells in *Foxo*-knockdown shrimp. **(D’)** The results of statistical analysis of three replicates for panel D. **(E)** The nuclear translocation of FOXO protein in the hemocytes from *Foxo*-knockdown shrimp at 2 h post *V*. *anguillarum* challenge analyzed by fluorescent immunocytochemical assay. Scale bar = 20 μm. **(E’)** Statistical analysis of FOXO nuclear translocation. WCIF ImageJ software was used to analyze co-localization by detecting the fluorescence intensity ratio of anti-FOXO (green) and DAPI-stained nuclei (blue) in hemocytes.
(TIF)

S4 Fig Nucleotide sequence of *Alf-E1* promoter. The FOXO binding sites of *Alf-E1* genomic sequence were marked. with green. Transcriptional start site marked with red.
(TIF)

S5 Fig RAB5 and RAB7 were upregulated in shrimp challenged with *V*. *anguillarum* and play important role in shrimp antibacterial defense. **(A, B)** The mRNA expression patterns of *Rab5* (A) and *Rab7* (B), as analyzed by qPCR. **(C, D)** Expression patterns of RAB5 and RAB7 proteins was analyzed using western blotting. The results of statistical analysis of three replicates are shown in the lower panels of (C) and (D). The relative expression levels of RAB5 or RAB7 normalized to that of β-actin were expressed as the mean ± SD, and the value of the control shrimp was set as 1. **(E)** Efficiency of *Rab5* and *Rab7* RNAi, as determined using western blotting and qPCR. **(F)** The survival rate of shrimp after knockdown of *Rab5* or *Rab7* following *V*. *anguillarum* infection. The survival rate of each group was calculated and the survival curves are presented as Kaplan–Meier plots.
(TIF)

S6 Fig FOXO promotes the expression RAB5 and RAB7 in intestine. (**A**) The mRNA expression level of the *Rab5* and *Rab7* in intestine of *Foxo*-RNAi shrimp with and without bacterial infection determined by qPCR. (**B**) The protein expression level of the RAB5 and RAB7 in intestine of the shrimp determined by western blotting. (**B’**), The results of statistical analysis of three replicates of panel (B).
(TIF)
