# Correction: FOXO regulates the expres sion of antimicrobial peptides and promotes phagocytosis of hemocytes in shrimp antibacterial immunity

**DOI:** 10.1371/journal.ppat.1013778

**Published:** 2025-12-16

**Authors:** Cang Li, Pan-Pan Hong, Ming-Chong Yang, Xiao-Fan Zhao, Jin-Xing Wang

The Data Availability statement for this article is incorrect. The authors have provided the data link DOI: 10.6084/m9.figshare.30452849.
