# Supplementary figures and images for: FOXO regulates the expression of antimicrobial peptides and promotes phagocytosis of hemocytes in shrimp antibacterial immunity
Source: PLoS Pathog. 2021 Apr 2;17(4):e1009479. doi: 10.1371/journal.ppat.1009479 (PMC8046353; doi:10.1371/journal.ppat.1009479)

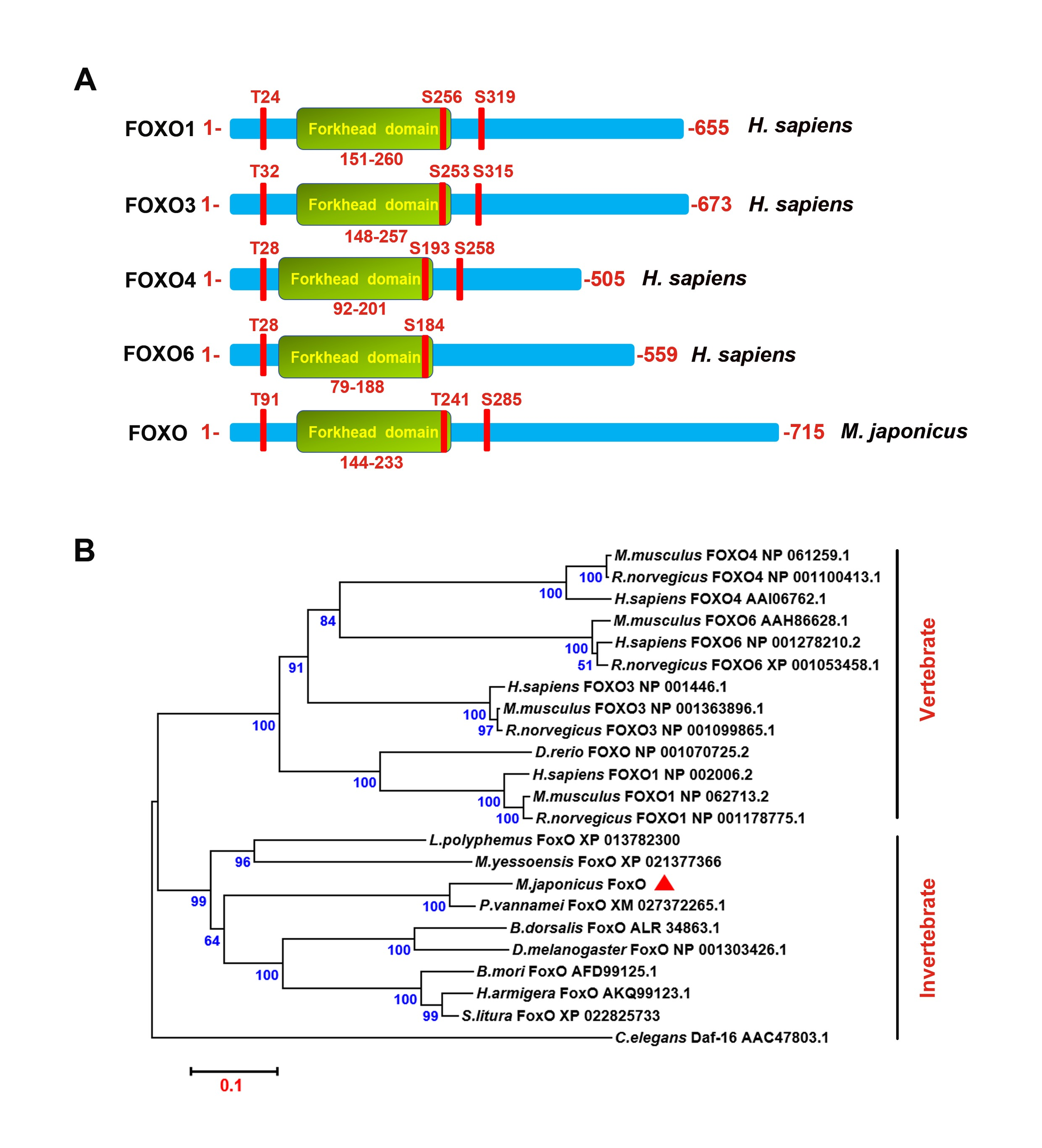

Supplement: S1 Fig — (A) Comparison of the FOXO domain architectures and AKT/PKB phosphorylation sites between M. japonicus and Homo sapiens. The FOXO amino acid sequence of Homo sapiens were obtained from GenBank (FOXO1, GenBank accession number: AAH70065.3; FOXO3, AAH68552.1; FOXO4, AAI06762.1; FOXO6, ARQ84049.1). (B) Phylogenetic tree of FOXOs from different species. The FOXO sequences of different species were obtained from GenBank, and the NJ tree was established using MEGA 6.0. The results were repeated 1000 times by bootstrapping. FOXO of M. japonicus is marked by a red triangle. (TIF) [file ppat.1009479.s001.tif]

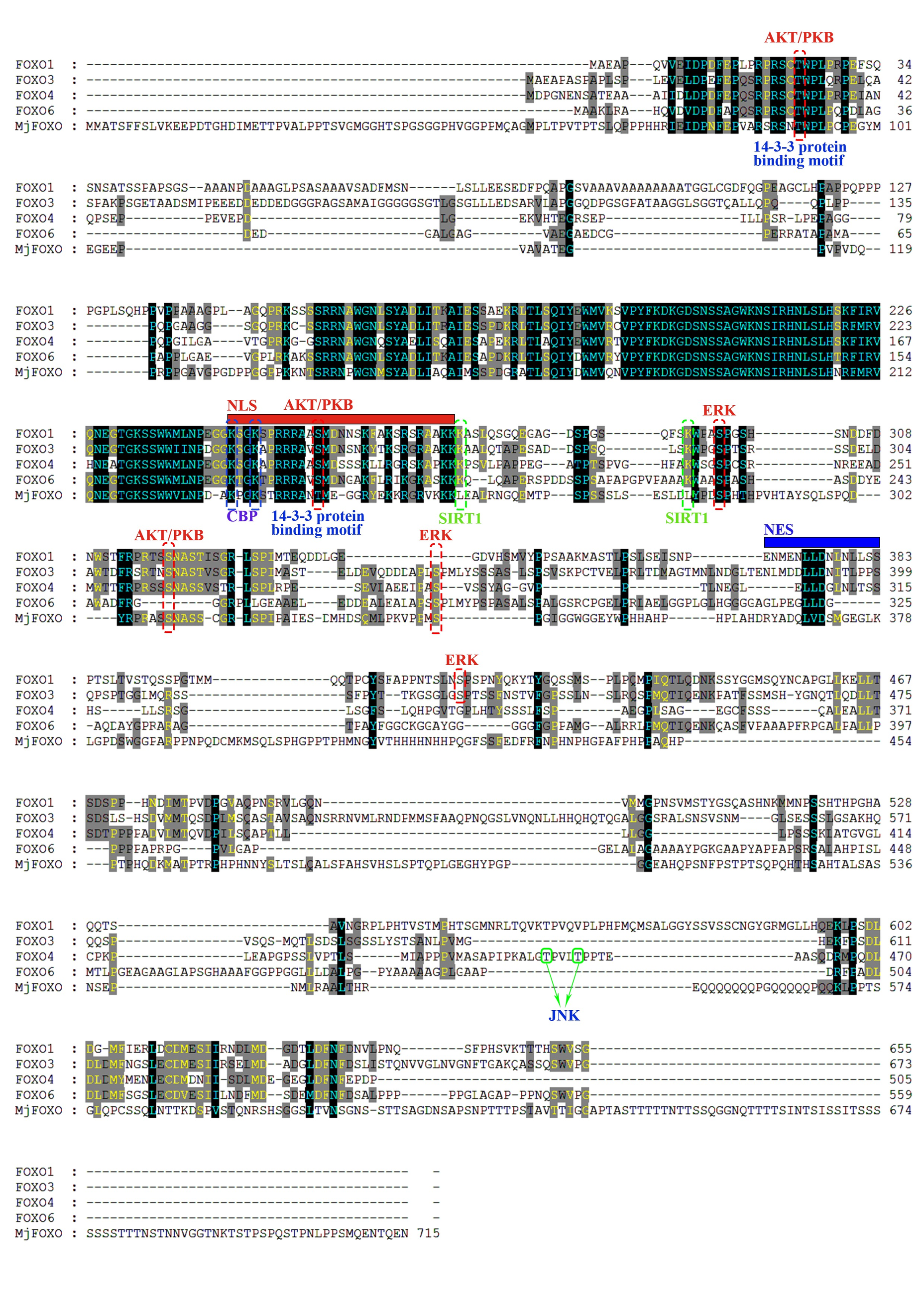

Supplement: S2 Fig — The posttranslational modification sites of FOXOs are shown in the alignment following previous reports on FOXOs. NLS: nuclear localization signal; NES: nuclear export sequence. CBP: cyclic-AMP responsive element binding (CREB)-binding protein; JNK: c-JUN N-terminal kinase; ERK: extracellular regulated protein kinase; SIRT1: Sirtuin1. (TIF) [file ppat.1009479.s002.tif]

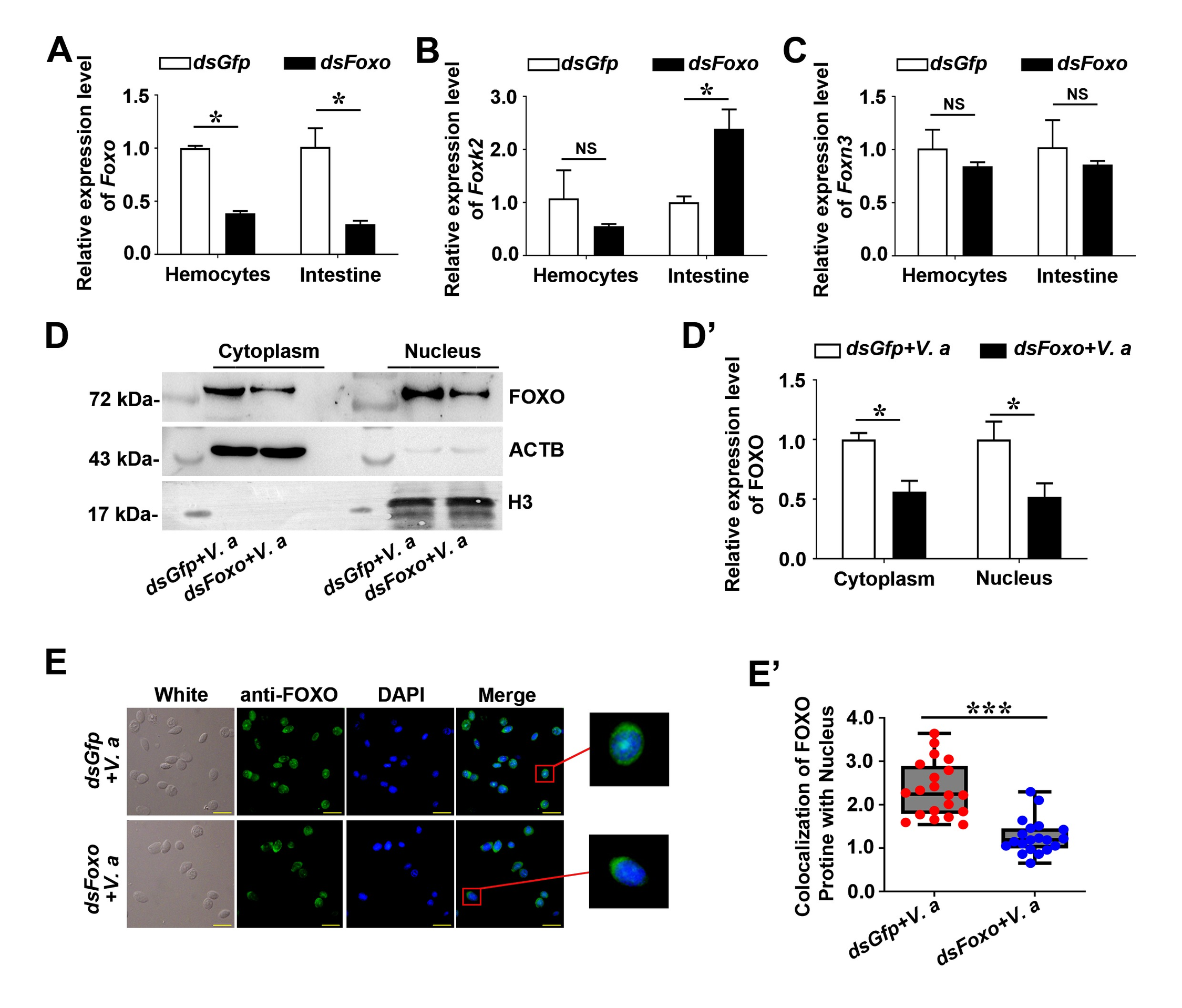

Supplement: S3 Fig — (A) Efficiency of Foxo-RNAi in hemocytes and intestines analyzed using qPCR. (B) mRNA expression of Foxk2 in Foxo-RNAi shrimp detected using qPCR. (C) mRNA expression of Foxn3 in Foxo-RNAi shrimp analyzed using qPCR. (D) Western blotting analysis of FOXO protein in the cytoplasm and nuclei of intestine cells in Foxo-knockdown shrimp. (D’) The results of statistical analysis of three replicates for panel D. (E) The nuclear translocation of FOXO protein in the hemocytes from Foxo-knockdown shrimp at 2 h post V. anguillarum challenge analyzed by fluorescent immunocytochemical assay. Scale bar = 20 μm. (E’) Statistical analysis of FOXO nuclear translocation. WCIF ImageJ software was used to analyze co-localization by detecting the fluorescence intensity ratio of anti-FOXO (green) and DAPI-stained nuclei (blue) in hemocytes. (TIF) [file ppat.1009479.s003.tif]

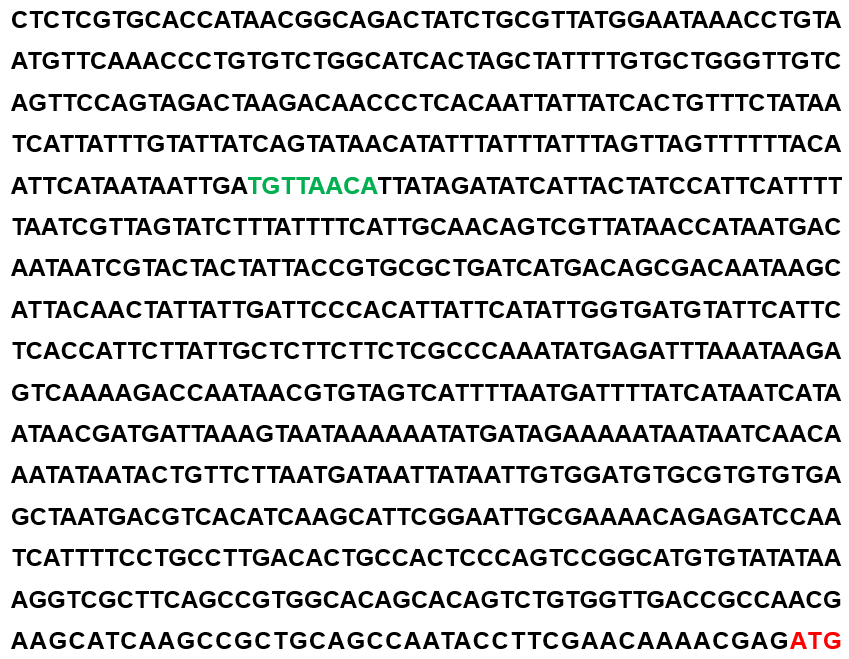

Supplement: S4 Fig — The FOXO binding sites of Alf-E1 genomic sequence were marked. with green. Transcriptional start site marked with red. (TIF) [file ppat.1009479.s004.tif]

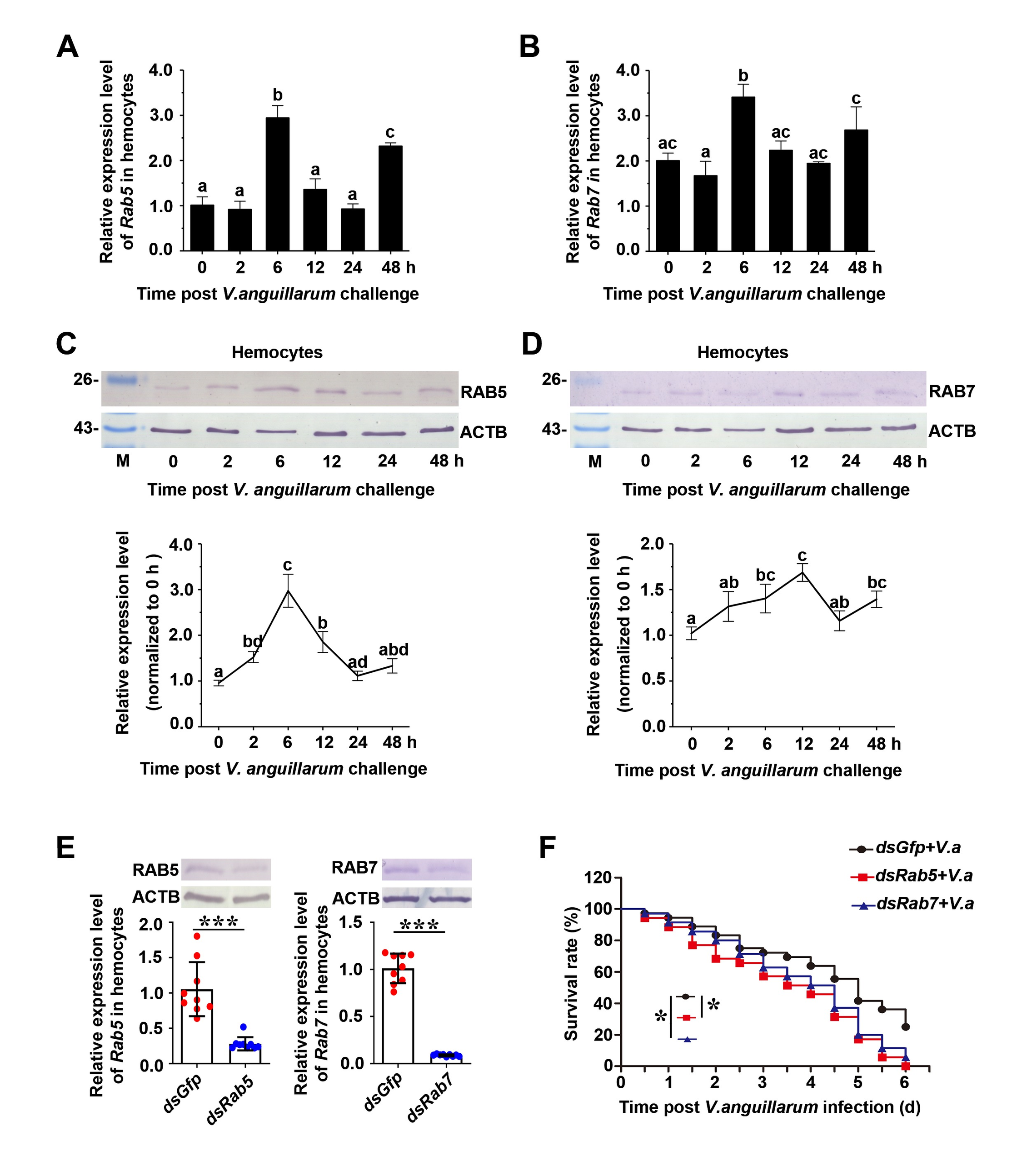

Supplement: S5 Fig — (A, B) The mRNA expression patterns of Rab5 (A) and Rab7 (B), as analyzed by qPCR. (C, D) Expression patterns of RAB5 and RAB7 proteins was analyzed using western blotting. The results of statistical analysis of three replicates are shown in the lower panels of (C) and (D). The relative expression levels of RAB5 or RAB7 normalized to that of β-actin were expressed as the mean ± SD, and the value of the control shrimp was set as 1. (E) Efficiency of Rab5 and Rab7 RNAi, as determined using western blotting and qPCR. (F) The survival rate of shrimp after knockdown of Rab5 or Rab7 following V. anguillarum infection. The survival rate of each group was calculated and the survival curves are presented as Kaplan–Meier plots. (TIF) [file ppat.1009479.s005.tif]

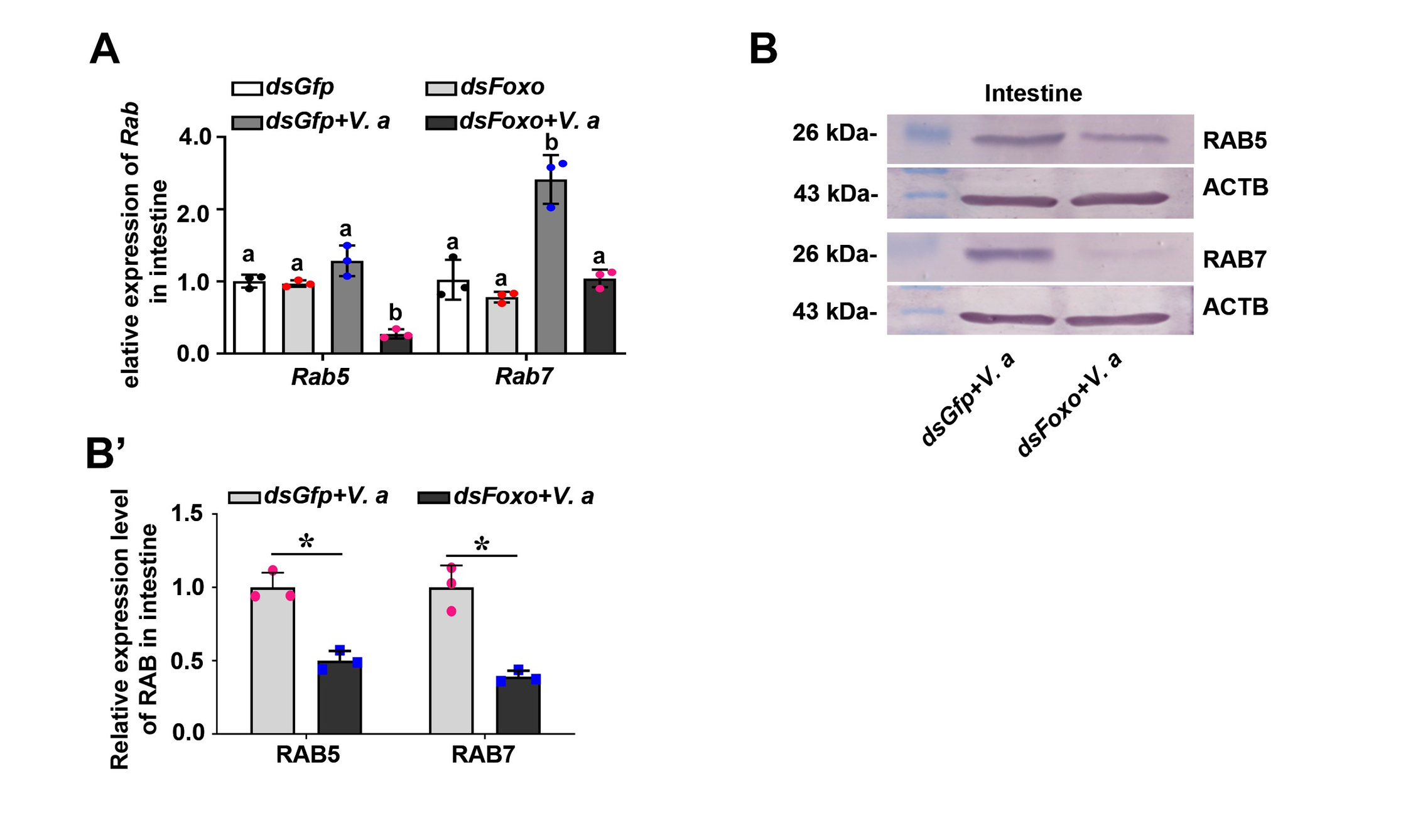

Supplement: S6 Fig — (A) The mRNA expression level of the Rab5 and Rab7 in intestine of Foxo-RNAi shrimp with and without bacterial infection determined by qPCR. (B) The protein expression level of the RAB5 and RAB7 in intestine of the shrimp determined by western blotting. (B’), The results of statistical analysis of three replicates of panel (B). (TIF) [file ppat.1009479.s006.tif]
